# Differential Physio-Biochemical and Metabolic Responses of Peanut (*Arachis hypogaea* L.) under Multiple Abiotic Stress Conditions

**DOI:** 10.3390/ijms23020660

**Published:** 2022-01-08

**Authors:** Jaykumar Patel, Deepesh Khandwal, Babita Choudhary, Dolly Ardeshana, Rajesh Kumar Jha, Bhakti Tanna, Sonam Yadav, Avinash Mishra, Rajeev K. Varshney, Kadambot H. M. Siddique

**Affiliations:** 1 CSIR—Central Salt and Marine Chemicals Research Institute, Bhavnagar 364002, India; jaypatelbt14@gmail.com (J.P.); deepeshkhandwal999@gmail.com (D.K.); choudharybabita212@gmail.com (B.C.); dolly19patel@gmail.com (D.A.); rajeshkumarjha2588@gmail.com (R.K.J.); tanna.bhakti90@gmail.com (B.T.); sonam_yadav11@yahoo.com (S.Y.); 2 Academy of Scientific and Innovative Research, Ghaziabad 201002, India; 3 Gujarat Biotechnology Research Centre, Gandhinagar 382011, India; 4 Centre of Excellence in Genomics & Systems Biology, International Crops Research Institute for the Semi-Arid Tropics (ICRISAT), Hyderabad 502324, India; rajeev.varshney@murdoch.edu.au; 5 The UWA Institute of Agriculture, UWA School of Agriculture and Environment, The University of Western Australia, Perth, WA 6001, Australia; 6 State Agricultural Biotechnology Centre, Centre for Crop and Food Innovation, Food Futures Institute, Murdoch University, Murdoch, WA 6150, Australia

**Keywords:** cold, combined stress, drought, groundnut, heat, metabolomics, peanut, salinity

## Abstract

The frequency and severity of extreme climatic conditions such as drought, salinity, cold, and heat are increasing due to climate change. Moreover, in the field, plants are affected by multiple abiotic stresses simultaneously or sequentially. Thus, it is imperative to compare the effects of stress combinations on crop plants relative to individual stresses. This study investigated the differential regulation of physio-biochemical and metabolomics parameters in peanut (*Arachis hypogaea* L.) under individual (salt, drought, cold, and heat) and combined stress treatments using multivariate correlation analysis. The results showed that combined heat, salt, and drought stress compounds the stress effect of individual stresses. Combined stresses that included heat had the highest electrolyte leakage and lowest relative water content. Lipid peroxidation and chlorophyll contents did not significantly change under combined stresses. Biochemical parameters, such as free amino acids, polyphenol, starch, and sugars, significantly changed under combined stresses compared to individual stresses. Free amino acids increased under combined stresses that included heat; starch, sugars, and polyphenols increased under combined stresses that included drought; proline concentration increased under combined stresses that included salt. Metabolomics data that were obtained under different individual and combined stresses can be used to identify molecular phenotypes that are involved in the acclimation response of plants under changing abiotic stress conditions. Peanut metabolomics identified 160 metabolites, including amino acids, sugars, sugar alcohols, organic acids, fatty acids, sugar acids, and other organic compounds. Pathway enrichment analysis revealed that abiotic stresses significantly affected amino acid, amino sugar, and sugar metabolism. The stress treatments affected the metabolites that were associated with the tricarboxylic acid (TCA) and urea cycles and associated amino acid biosynthesis pathway intermediates. Principal component analysis (PCA), partial least squares-discriminant analysis (PLS-DA), and heatmap analysis identified potential marker metabolites (pinitol, malic acid, and xylopyranose) that were associated with abiotic stress combinations, which could be used in breeding efforts to develop peanut cultivars that are resilient to climate change. The study will also facilitate researchers to explore different stress indicators to identify resistant cultivars for future crop improvement programs.

## 1. Introduction

The frequency and severity of abiotic stresses are increasing due to climate change and global warming [1]. Researchers generally study a single stressor to evaluate the mechanism or effect on plants. However, multiple abiotic and biotic stresses can simultaneously affect plants under field conditions [2]. Standard laboratory conditions that are often used for plant science research significantly differ from the field, so it is difficult to associate output results from an individual stress study to field conditions. Abiotic stresses can lead to the production of excess reactive oxygen species (ROS), mainly in chloroplasts, mitochondria, and peroxisomes, with detrimental effects on signaling behavior [3,4]. Compared to individual stresses, combined abiotic stresses respond differently to ROS production through the differential production of enzymatic and non-enzymatic antioxidants in plant cells, resulting in a unique ROS signature and acclimation response via modifications to the signaling pathway [2,5,6]. Plants under combined abiotic stresses also differ from those that are under individual stresses for photosynthesis, stomatal regulation, and water use efficiency (WUE) [7,8]. For example, the net photosynthesis rate of soybean decreased more under combined water deficit and heat stress than individual stresses due to reduced CO_2_ availability, lower relative water content (RWC), and higher leaf temperature [9,10]. Similarly, WUE (directly linked to stomatal opening or closing) decreased in most studies under different stress combinations [11,12]. Combined abiotic stresses significantly reduce crop productivity and yield by affecting plant reproductive processes [13]. Recent studies in maize and wheat showed that combined abiotic stresses considerably decreased crop yield by reducing stigma functionality and kernel abortion [14,15].

Metabolomics is an emerging technology in plant biology representing data output from gene expression, protein interaction, and pathway regulations. Untargeted metabolic profiling of plant samples under different abiotic stresses is a new dimension for plant metabolic pathway and signaling research [16]. Metabolomics of rice flowering organs under combined drought and heat stress revealed that ribitol, pyruvic acid, and succinic acid significantly correlated with yield and the chalky grain fraction of seeds. In addition, the combined stress significantly increased the arbutin levels in flag leaves; this glycoside has strong antioxidant and membrane-stabilizing properties [17]. Eucalyptus under combined heat and drought stress significantly decreased WUE and differential metabolite accumulation compared to individual stresses [18]. Organic acids and carbohydrates, such as succinate, malate, quinate, glycerate, mannose, and galactose, significantly decreased, while most amino acids, including aspartate, glutamate, aspargine, valine, leucine, isoleucine, proline threonine, lysine, and histidine, significantly increased under combined stress [18]. Metabolomics can also be used to identify quantitative trait loci (QTL) and markers under various stresses, such as those that are related to the antioxidant enzyme system that was identified in barley and potato under combined drought and heat stress [19,20].

Peanut or groundnut (*Arachis hypogaea* L.) is a major oilseed legume that is grown in subtropical and tropical regions [21]. Peanut is a rich source of oil (40–60%), protein (10–20%), carbohydrates, vitamins, minerals, antioxidants, and monounsaturated fatty acids, and a source of medicinally important compounds [22,23]. India is the world’s largest edible oil consumer and peanut oil is the third-most consumed edible oil in India after palm and soybean oil. Abiotic stresses such as drought, salinity, and heat frequently affect peanut production, as it mainly grows in subtropical and tropical regions. Therefore, efforts are underway to improve abiotic stress tolerance in peanut [21,24,25,26]. Some studies have investigated the effect of abiotic stresses on physiological, biochemical, and metabolic changes in peanut [22,23,27,28]. One study evaluated the physiological and biochemical characteristics of ancestral peanut species under drought stress, revealing *Arachis ipaensis* as the most drought-tolerant due to higher solute accumulation in the roots than the other varieties [22]. A metabolomics study reported that drought-tolerant peanut varieties accumulated important polyamines and polyphenols such as agmatine, cadaverine, syringic acid, and vanillic acid under stress [23]. There were also two studies that reported stress-specific metabolite accumulation in peanut [27,28].

This study investigates the physio-biochemical response of peanut under different individual (salinity, drought, heat, and cold) and combined abiotic stresses (S-D: salinity and drought, S-H: salinity and heat, S-C: salinity and cold, D-H: drought and heat, D-C: drought & cold, S-D-H: salinity, drought & heat, and S-D-C: salinity, drought and cold) by measuring indicator parameters of physiology, biochemical processes, and osmolytes. The metabolic profiles were studied using GC-MS (gas chromatography–mass spectrometry), multivariate correlation analysis, pathway enrichment analysis, heatmaps, and partial least square analysis.

## 2. Results

### 2.1. Biochemical Status of Plants under Different Stress Conditions

**Figure 1:**
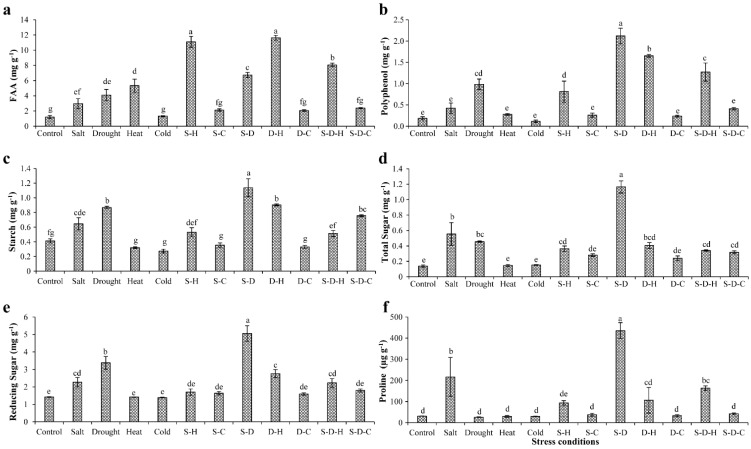
Biochemical status of plants under different stress conditions. Estimation of (**a**) free amino acids, (**b**) polyphenols, (**c**) starch, (**d**) total sugars, (**e**) reducing sugars, and (**f**) proline in peanut under individual and combined stresses (S-D: salinity and drought, S-H: salinity and heat, S-C: salinity and cold, D-H: drought and heat, D-C: drought and cold, S-D-H: salinity, drought, and heat, and S-D-C: salinity, drought, and cold). The data are the mean ± SE; different letters indicate significant differences at *p* < 0.05.

In peanut, the various stresses adversely affected the biochemical constituents, including sugars, starch, amino acids, and polyphenols (Figure 1). Free amino acids (FAA) increased in all the stress treatments except for individual cold stress and cold-containing combined stresses, relative to the control (unstressed) plants (Figure 1a). The maximum increases in FAA occurred under D-H (11.64 ± 0.31 mg g^−1^, 9.65-fold) and S-H (11.10 ± 0.73 mg g^−1^, 9.2-fold) stress followed by S-D-H (8.06 ± 0.23 mg g^−1^, 6.68-fold) and heat (5.33 ± 0.85 mg g^−1^, 4.4-fold) stress, compared to the unstressed plants (1.20 ± 0.20 mg g^−1^). In contrast, the smallest increments in FAA occurred in plants that were grown under combined stress that included cold [S-C (2.12 ± 0.15 mg g^−1^, 1.76-fold), D-C (2.05 ± 0.11 mg g^−1^, 1.70-fold), and S-D-C (2.38 ± 0.07 mg g^−1^, 1.97-fold)].

The polyphenol contents significantly increased in individual drought stress (0.98 ± 0.12 mg g^−1^, 5.32-fold) and S-D (2.12 ± 0.18 mg g^−1^, 11.45-fold), D-H (1.65 ± 0.03 mg g^−1^, 8.92-fold), S-H (0.81 ± 0.24 mg g^−1^, 4.39-fold), and S-D-H (1.27 ± 0.21 mg g^−1^, 6.87-fold) stresses compared to the control plants (Figure 1b). Similarly, the starch content significantly increased under individual drought stress (0.86 ± 0.01 mg g^−1^, 2.1-fold) and combined stress that included drought [S-D (1.13 ± 0.12 mg g^−1^, 2.75-fold), D-H (0.90 ± 0.01 mg g^−1^, 2.18-fold), S-D-C (0.75 ± 0.01 mg g^−1^, 1.83-fold)] compared to the unstressed plants (0.41 ± 0.02 mg g^−1^) (Figure 1c). A similar pattern occurred for the total and reducing sugar concentrations (Figure 1d,e). The sugar contents significantly increased under individual drought stress (total sugars, 1.16 ± 0.07 mg g^−1^, 8.37-fold; reducing sugars, 5.06 ± 0.44 mg g^−1^, 3.56-fold) compared to the control plants (total sugars, 0.13 ± 0.01 mg g^−1^; reducing sugars, 1.42 ± 0.01 mg g^−1^).

Proline provides abiotic stress tolerance to plants by modulating osmotic adjustment [29]. The elevated proline content occurred in plants that were grown under individual salt stress and combined stresses that included salt (Figure 1f), more so for S-D (435.20 ± 37.70 µg g^−1^, 14.16-fold) compared to the control (30.72 ± 0.47 µg g^−1^). The proline concentrations significantly increased by about 7.04- and 5.32-fold under salt (216.40 ± 91.23 µg g^−1^) and S-D-H (163.54 ± 10.41 µg g^−1^) stresses, respectively, compared to the control plants, with no significant changes in the other treatments.

### 2.2. Physiological Status of Plants under Different Stress Treatments

**Figure 2:**
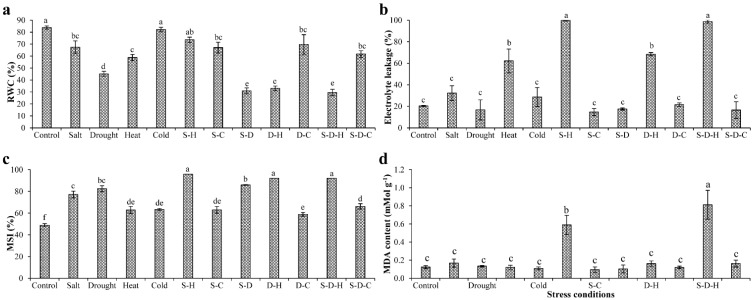
Physiological status of plants under different stress conditions. The estimation of (**a**) relative water content (RWC), (**b**) electrolyte leakage, (**c**) membrane stability index (MSI), and (**d**) lipid peroxidation (MDA content) in peanut plants under different individual and combined stresses (S-D: salinity and drought, S-H: salinity and heat, S-C: salinity and cold, D-H: drought and heat, D-C: drought and cold, S-D-H: salinity, drought, and heat, and S-D-C: salinity, drought, and cold). The data are the mean ± SE; different letters indicate significant differences at *p* < 0.05.

The relative water content of all the plant samples significantly decreased in all stress treatments except for cold and S-H, where it did not change significantly (82.13 ± 1.86%) compared to the unstressed plants (83.86 ± 1.34%). RWC substantially decreased in plants under S-D (30.90 ± 2.54%), D-H (33.00 ± 1.90%), and S-D-H (29.59 ± 2.60%) stress compared to the unstressed plants (83.86 ± 1.34%), with a notable decline for individual drought stress (45.10 ± 2.19%), followed by the remaining stresses [salt (67.34 ± 5.29%), heat (58.76 ± 2.56%), S-H (73.59 ± 2.21%), S-C (67.06 ± 4.45%), D-C (69.73 ± 8.10%), S-D-C (61.67 ± 2.75%)] (Figure 2a). Cold stress ameliorated leaf RWC when combined with drought stress, increasing from 45.10 ± 2.19% under individual drought stress to 69.73 ± 8.10% under D-C.

The individual and combined heat stress significantly increased EL (Figure 2b), particularly under S-H (99.88 ± 0.59%) and S-D-H (98.71 ± 1.50%), about five-fold higher than the unstressed plants (20.36 ± 0.50%). Similarly, about three-fold increases in EL occurred under heat (62.18 ± 11.00%) and D-H stress (68.39 ± 1.54%) compared to the control. Interestingly, EL did not significantly change under salt, drought, cold, S-C, S-D, D-C, or S-D-C stress. The membrane stability increased in all the abiotic stress treatments compared to the control (Figure 2c).

Lipid peroxidation (MDA content) did not significantly change in most stress treatments except for S-H and S-D-H (Figure 2d), which significantly increased [S-H (0.58 ± 0.10 mM g^−1^) and S-D-H (0.81 ± 0.15 mM g^−1^)] compared to the control (0.12 ± 0.01 mM g^−1^) (Figure 2d).

The total chlorophyll content decreased negligibly under S-H (0.23 ± 0.03 mg g^−1^ FW) and S-C (0.21 ± 0.02 mg g^−1^ FW) stress compared to the unstressed plants (0.26 ± 0.01 mg g^−1^ FW). In contrast, the total chlorophyll contents significantly increased under the other combined stresses [S-D (0.44 ± 0.02 mg g^−1^ FW), D-H (0.37 ± 0.07 mg g^−1^ FW), D-C (0.41 ± 0.005 mg g^−1^ FW), S-D-H (0.48 ± 0.08 mg g^−1^ FW), S-D-C (0.44 ± 0.006 mg g^−1^ FW)] compared to the control (Supplementary Figure S1). The total chlorophyll, chlorophyll *a*, chlorophyll *b*, and carotene did not significantly change under the individual stresses.

### 2.3. Antioxidant Enzyme Assays, Transcript Expression Analysis of Encoding Genes, and In Vivo ROS Localization

**Figure 3:**
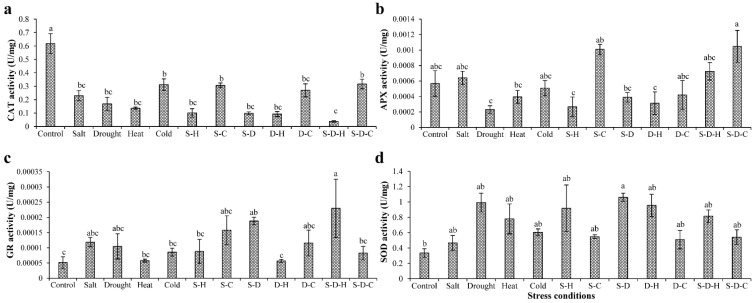
Antioxidant enzyme assays. The estimation of (**a**) catalase (CAT), (**b**) ascorbate peroxidase (APX), (**c**) glutathione reductase (GR), and (**d**) superoxide dismutase (SOD) activities in peanut plants under different individual and combined stresses (S-D: salinity and drought, S-H: salinity and heat, S-C: salinity and cold, D-H: drought and heat, D-C: drought and cold, S-D-H: salinity, drought, and heat, and S-D-C: salinity, drought, and cold). The data are the mean ± SE; different letters indicate significant differences at *p* < 0.05.

Quantitative RT-PCR and biochemical enzyme assays were used to analyze the antioxidant activities in peanut plants under various abiotic stresses (Supplementary Figure S2). The transcripts of SOD (Figure S2a) and APX (Figure S2b)-encoding genes significantly increased in plants under abiotic stress compared to the unstressed plants, with maximum increases for SOD (9-fold) under salt stress and APX (11-fold) under D-H stress. The CAT expression transcript (Figure S2c) decreased under all the stress conditions except for individual cold stress, with a maximum 20-fold reduction under S-D. Similarly, the GR transcript (Figure S2d) increased in all the stress treatments except for drought and S-D stress. The antioxidant encoding enzymes changed the least under individual cold stress and the combined stresses that included cold. In contrast, heat stress combined with salt or drought stress showed the maximum differential expression of antioxidant encoding transcripts. Similar results occurred for the biochemical antioxidant enzyme assays of CAT, SOD, and GR, which showed enzyme activities that were similar to their respective transcript profiling (Figure 3). However, APX activity increased under salt (12%), S-C (77%), S-D-C (84%), and S-D-H (27%) stress but decreased in all other stress treatments compared to the control. CAT activity declined, and SOD and GR activity increased in all the stress treatments compared to the control.

**Figure 4:**
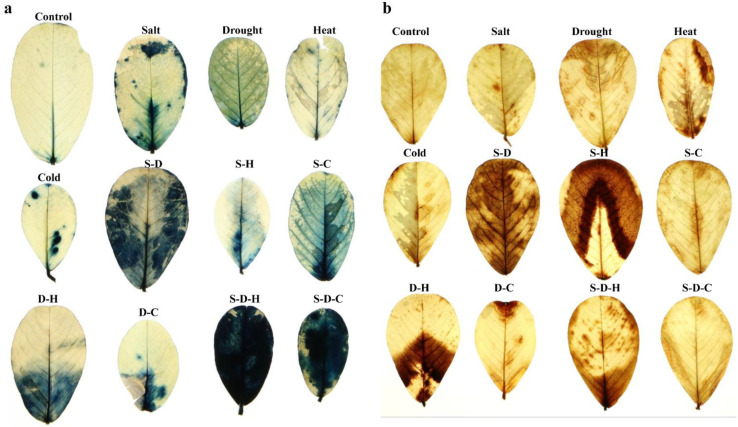
Qualitative histochemical assay of peanut leaves. (**a**) NBT assay for identifying the accumulation of free oxygen radicals, and (**b**) DAB assay for identifying the accumulation of H_2_O_2_ in peanut leaves under different individual and combined stresses (S-D: salinity and drought, S-H: salinity and heat, S-C: salinity and cold, D-H: drought and heat, D-C: drought and cold, S-D-H: salinity, drought, and heat, and S-D-C: salinity, drought, and cold).

NBT (nitro-blue tetrazolium) and DAB (3,3-diaminobenzidine) staining qualitative assays were undertaken to analyze the endogenous peroxide and free oxygen radicals that were generated in the leaf tissues under different stress conditions (Figure 4). The control leaf samples had nominal staining, indicating minimal ROS production. The leaves that were under S-D-H stress had the most intense blue stain (NBT), indicating a high generation of free oxygen radicals, followed by salt, S-D, S-C, and S-D-C stress and relatively low stain intensity for drought, heat, cold, and D-C stress. The DAB stain results showed that individual stresses did not induce much H_2_O_2_, while some combined stresses (S-D, S-H, D-H, and S-D-H) accumulated high amounts of brown DAB precipitates, suggesting high H_2_O_2_ production. Overall, individual heat stress and combined stress that included heat produced high amounts of peroxide radicals, while cold stress ameliorated the effects of the other stresses by preventing peroxide radical production.

Dose-dependent total antioxidant and scavenging activities were observed under different stress conditions (Supplementary Figure S3). The total antioxidant and scavenging activities significantly decreased under drought stress (Supplementary Figure S3). The total phenolics content increased under D-C stress but decreased under individual drought stress compared to the control. Interestingly, negligible changes in total flavonoid content occurred under any abiotic stress (Supplementary Figure S3).

### 2.4. Ion Content Analysis of Plants Subjected to Different Stress Conditions

The sodium and potassium ion contents of the treated plant samples were recorded by ICP-OES (Supplementary Figure S4). The potassium content significantly increased under cold (0.61 ± 0.003%wt), S-C (0.52 ± 0.005%wt), S-D (1.61 ± 0.006%wt), D-H (0.48 ± 0.002%wt), D-C (0.68 ± 0.01%wt), and S-D-C (0.53 ± 0.01%wt) stress compared to the control (0.44 ± 0.006%wt). A lower K^+^ content occurred under heat (0.20 ± 0.001%wt), S-H (0.16 ± 0.001%wt), and S-D-H (0.15 ± 0.001% wt) stress compared to the control. In contrast, Na^+^ content increased under salt (0.17 ± 0.008%wt), S-H (0.14 ± 0.001%wt), D-C (0.08 ± 0.001%wt), and S-D-H (0.08 ± 0.001%wt) stressed but decreased under cold (0.03 ± 0.001%wt), S-D (0.01 ± 0.001%wt), D-H (0.01 ± 0.001%wt), and S-D-C (0.03 ± 0.005%wt) stress compared to the control plants (0.04 ± 0.001%wt). The Na^+^/K^+^ ratio increased significantly under salt (0.52), heat (0.18), S-H (0.88), and S-D-H (0.42) stress compared to the control (0.11) but significantly decreased under drought (0.09), cold (0.05), S-C (0.08), S-D (0.01), D-H (0.03), and S-D-C (0.05) stress. The results indicate that the Na^+^/K^+^ ratio increased under salt- and heat-related stresses but decreased under cold and drought stresses.

### 2.5. Multivariate Correlation Analysis

**Figure 5:**
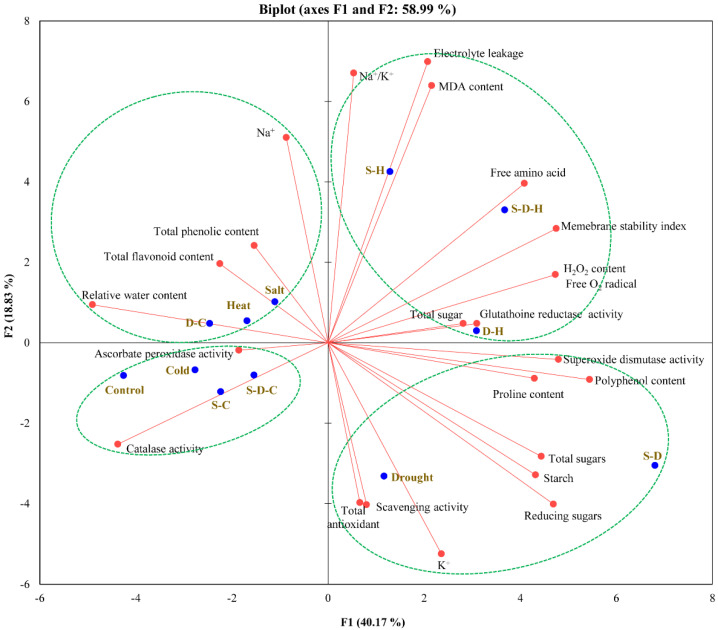
Correlation analysis (PCA biplot) between the different individual and combined abiotic stresses and physio-biochemical parameters in peanut plants (S-D: salinity and drought, S-H: salinity and heat, S-C: salinity and cold, D-H: drought and heat, D-C: drought and cold, S-D-H: salinity, drought, and heat, and S-D-C: salinity, drought, and cold).

A principal component analysis (PCA) of the studied biochemical and physiological variables (excluding metabolomics data) was undertaken for all the abiotic stress treatments. The biplot shows a total variability of 58.99%, with F1 and F2 accounting for 40.17% and 18.83% of the variability, respectively, between the physio-biochemical parameters and abiotic stress treatments (Figure 5). Squared cosines of the control (0.785), heat (0.315), cold (0.717), S-C (0.334), S-D (0.719), D-H (0.501), and S-D-H (0.401) stress were significantly higher than the other stresses indicating a greater contribution in the PCA, whereas the physio-biochemical parameters such as free oxygen radicals (7.37%), H_2_O_2_ content (7.37%), SOD activity (7.57%), polyphenol content (9.75%), reducing sugar concentration (7.24), RWC (7.90%), and MSI (7.43%) were significant contributors to the PCA. Figure 5 shows that cold, S-C, and S-D-C stress positively correlated with the control, while S-H, S-D-H, and D-H negatively correlated with the control. The variables including RWC, Na^+^ concentration, TPC (total phenolic content), and TFC (total flavonoid content) positively correlated with D-C, salt, and heat stress. Similarly, the total antioxidant, radical scavenging, total sugars, reducing sugars, starch, polyphenol, proline concentration, and SOD activity positively correlated with drought and S-D stress. Catalase and APX activities correlated with cold, S-C, and S-D-C stress. Similarly, EL, MSI, lipid peroxidation, FAA, H_2_O_2_, free oxygen radicals, total chlorophyll, and GR activity positively correlated with S-H, S-D-H, and D-H stress. Pearson’s correlation matrix revealed correlations among the different variables at a 95% confidence interval (Supplementary Table S1). The proline concentration positively correlated with the total sugars (0.926; R^2^ = 0.857), followed by the reducing sugars (0.800; R^2^ = 0.640), oxygen radicals (0.714; R^2^ = 0.510), H_2_O_2_ concentration (0.714; R^2^ = 0.510), polyphenols (0.740; R^2^ = 0.548), potassium ion (0.659; R^2^ = 0.434), and starch (0.658; R^2^ = 0.433). In contrast, CAT activity (−0.428; R^2^ = 0.183) and RWC (−0.550; R^2^ = 0.302) had negative but not significant correlations with the proline concentration. As expected, lipid peroxidation positively correlated with EL (0.832; R^2^ = 0.692), and Na^+^/K^+^ ratio (0.702; R^2^ = 0.493) and negatively correlated with CAT activity (−0.474; R^2^ = 0.225) and K^+^ concentration (−0.457; R^2^ = 0.208). Similarly, the H_2_O_2_ concentration negatively correlated with CAT activity (−0.565; R^2^ = 0.319). In contrast, GR activity positively correlated with free oxygen radicals (0.595; R^2^ = 0.354), H_2_O_2_ concentration (0.595; R^2^ = 0.354), and proline concentration (0.578; R^2^ = 0.334).

### 2.6. Metabolite Profiling of Plants under Different Stress Combinations

The GC-MS identified 160 metabolites, including 20 amino acids, 8 fatty acids, 33 organic acids, 28 sugars, 3 sugar acids, 4 sugar alcohols, and 64 other metabolites (Table 1).

Collectively, 23 metabolites were detected for all individual stresses, of which the concentration of 5 decreased, 7 increased, and 11 did not change. Amino acids were prevalent among those that decreased, including phenylalanine (−438-fold), glycine (−11-fold), proline (−4-fold), and threonine (−3-fold). Organic acids and sugars were prevalent among those that increased, including palmitic acid (4-fold), xylopyranose (3-fold), and pentanedioic acid (3-fold). Under salt stress, amino acid concentrations decreased, such as phenylalanine (−19-fold) and glycine (−11-fold), while the concentrations of metabolites that are involved in the TCA cycle and some organic acids increased significantly [citric acid (7-fold), tartaric acid (2-fold), propanedioic acid (5-fold), and butenedioic acid (4-fold)]. The phenylalanine concentration decreased significantly under drought (−490-fold), heat (−57-fold), and cold (−1187-fold) stress. Similarly, glycine decreased under drought (−32-fold) and cold (−1186-fold) stress but increased under heat stress (10-fold). In contrast, palmitic acid increased under all individual stresses [salt (12-fold), drought (4-fold), heat (3-fold), and cold (5-fold)].

Collectively, 25 metabolites were detected across all combined stresses, of which the concentration of 12 decreased, 4 increased, and 9 did not change. The concentrations of citric acid (3-fold), silanol (2-fold), glutamic acid (2-fold), and glyceric acid (15-fold) increased while those of amino acids decreased [phenylalanine (−502-fold), lysine (−73-fold), tyrosine (−57-fold), leucine (−20-fold), glycine (−17-fold), asparagine (−7.25-fold), threonine (−4-fold)]. The hydroxyproline (proline derivative) concentrations increased under S-D (4-fold), D-H (6-fold), and S-D-H (10-fold) stress.

### 2.7. Principal Component Analysis, Partial Least Square Discriminant Analysis and Heatmap Analysis of Metabolites

The PCA was performed (Supplementary Figure S5) to identify correlations between the different individual and combined abiotic stresses and changes in metabolite accumulation. The biplot for the individual stresses shows 65.26% total variability, with PC1 contributing 35.99% and PC2 contributing 29.27%, and the control group significantly differed from the individual abiotic stresses at the 95% confidence interval. Of the 60 squared cosines, 20 metabolites were significantly higher and contributed to the principal components. Among the metabolites, isoleucine (4.28%), butenedioic acid (3.03%), glycolic acid (4.12%), aminobutanoic acid (3.790%), aconitic acid (4.12%), fructose (4.12%), ferulic acid (4.12%), caffeic acid (4.22%), and galactinol (4.12%) contributed the most to the principal components. The biplot also shows a significant correlation between the accumulation of proline under salt stress, valine and leucine under cold stress, isoleucine and fructose under drought stress, and citric acid and ketoadipic acid under heat stress. The biplot for the combined stresses shows 57.15% total variability, with PC1 contributing 36.77% and PC2 contributing 20.40%. The squared cosines of 28 metabolites were significantly high and contributed to the principal components. Propanedioic acid (4.08%), threonine (3.89%), phenylalanine (4.08%), asparagine (3.5%), and tryptophan (3.70%) contributed the most to the principal components. The combined stress biplot shows two distinct groups that significantly differed from the control. The first group correlated S-H, S-D, and S-D-H stress (salt as a common factor) with the accumulation of glyceric acid, caffeic acid, urea, isoleucine, tyrosine, and glycolic acid. The second group correlated S-C, D-C, and S-D-C stress (cold as a common factor) with the accumulation of glutamine, 2-methylalanine, psicofuranose, and shikimic acid.

The metabolomics data for peanut plants under different abiotic stresses were analyzed by partial least squares-discriminant analysis (PLS-DA) to understand the correlation between the changes in the metabolite concentration and the different abiotic stresses (Supplementary Figure S6). Multivariate regression was used and performed using the plsr function in the R pls package. Further classification and cross-validation of the metabolomics data was performed using the caret package of Metaboanalyst ver. 5.0. The variable importance in projection (VIP) score of the metabolites was calculated from the weighted sum of squares of the PLS loading against each treatment. Important features of metabolites with the highest VIP scores were plotted against the different treatments, and a 3D score plot for each treatment was predicted (Supplementary Figure S7). The VIP score plot showed that the individual stresses significantly changed the concentrations of pinitol, xylopyranose, phenylalanine, and proline, while the combined stresses significantly changed the concentrations of pinitol, malic acid, glutamic acid, silanol, and citric acid.

Metabolite heatmaps were generated with clustering features (metabolites) and variables (treatments) (Supplementary Figure S8). The results showed that the control had very low amino acid concentrations (proline, tyrosine, alanine, glycine, and tryptophan) compared to the individual stresses (drought, salt, heat, and cold). In contrast, the individual stresses significantly decreased the concentration of metabolites belonging to organic acids, fatty acids, and sugars (stearic acid, pentanedioic acid, ketoglutaric acid, tartaric acid, palmitic acid, xylopyranose, and melibiose). Proline, glycine, serine, sucrose, myo-inositol, and glyceric acid concentrations increased significantly under salt stress compared to the control. Similarly, phenylalanine, threonine, tryptophan, quininic acid, butanedioic acid, leucine, and valine increased under cold stress; 2-ketoadipic acid, malic acid, ethanolamine, citric acid, shikimic acid and glutamic acid increased under heat stress; and aconitic acid, glycolic acid, fructose, ferulic acid, galactinol, asparagine, and caffeic acid increased under drought stress compared to the control. A cluster analysis showed that, compared to the control, changes in the metabolite profile were more related under cold stress and most divergent under drought stress. The metabolite heatmap for the combined stresses showed that cold stresses (S-C, D-C, and S-D-C) showed the most similar changes in profiling compared to the control. In contrast, heat stresses (D-H, S-H and D-H) were distant to the control and showed the most divergent changes in metabolite profiling. The concentrations of fructose, psicofuranose, 2-methylalanine, shikimic acid, quininic acid, and glyceric acid increased under S-C, D-C, and S-D-C stress compared to the control. Similarly, glycolic acid, propanedioic acid, tryptophan, asparagine, phenylalanine, threonine, aconitic acid, and tyrosine accumulated under D-H, S-H, S-D, and S-D-H stress. In addition, the heatmap analysis revealed correlations for the selected metabolites under individual and combined stresses (Supplementary Figure S9).

### 2.8. Pathway Enrichment Analysis

**Figure 6:**
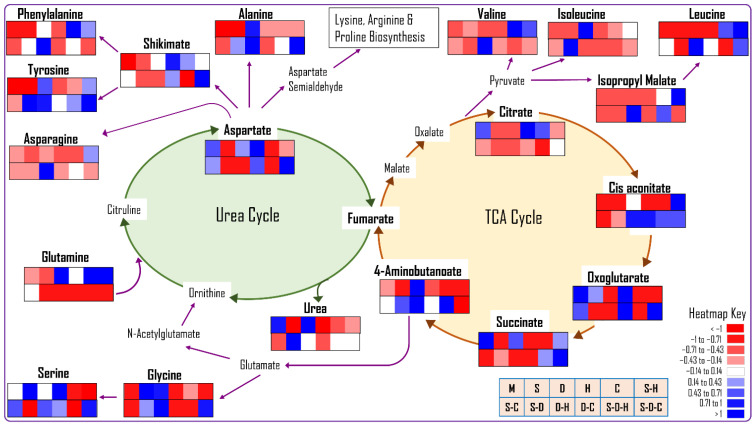
The differential accumulation of metabolites that are involved in the TCA and urea cycles and various amino acid biosynthesis pathways in peanut plants under different abiotic stresses (M: no stress, S: salinity, D: drought, H: heat, C: cold, S-D: salinity and drought, S-H: salinity and heat, S-C: salinity and cold, D-H: drought and heat, D-C: drought and cold, S-D-H: salinity, drought, and heat, S-D-C: salinity, drought, and cold).

The Venn diagrams for individual and/or combined stresses show exclusive and inclusive metabolite accumulation (Supplementary Figure S10), with 18 metabolites that were common to the individual stresses and the control. Further, 39, 7, 7, and 10 metabolites were exclusively detected under drought, salt, heat, and cold stress, respectively. For the combined stresses, 18, 9, 9, 16, 7, 42, and 24 metabolites were exclusively found under S-H, D-C, S-C, S-D, D-H, S-D-H, and S-D-C stress, respectively. The metabolites that were observed exclusively under abiotic stress (individual or combined stress) were used for the pathway enrichment analysis (Supplementary Figure S11). The pathway impact summary plots were generated by pathway topology analysis using the Arabidopsis KEGG metabolic pathway database. The large circle on the pathway impact plot indicates the highest impact and the darker color indicates significant changes in concentration. The individual and combined stresses significantly affected amino acid metabolism pathways, including aminoacyl-tRNA biosynthesis, valine, leucine and isoleucine metabolism, beta-alanine metabolism, tryptophan metabolism, phenylalanine metabolism, lysine metabolism, tyrosine metabolism, and arginine biosynthesis, along with the pathways of galactose metabolism, glucosinolate biosynthesis, pentose and glucuronate interconversion, pantothenate and CoA biosynthesis, indole alkaloid biosynthesis, tropane, piperidine and pyridine alkaloid biosynthesis, isoquinoline alkaloid biosynthesis, and pyrimidine metabolism. The fatty acid degradation, amino sugar metabolism, and nucleotide sugar metabolism pathways were least affected by the various abiotic stresses. Changes in the metabolite concentration from the interconnected TCA and urea cycles and amino acid biosynthesis are shown in the heatmap (Figure 6).

## 3. Discussion

Climate change and global warming have increased the severity and frequency of extreme climatic conditions. Plant biotechnology researchers often work on individual abiotic stresses, but plants usually experience multiple abiotic stresses at once or sequentially in the field, inducing different responses to individual stresses. Plants selectively change physio-biochemical properties and metabolic pathways to adjust to the adverse effects of these abiotic stresses. The combination of some abiotic stresses such as drought and heat may produce conflicting responses in plants. Plant adaptation to abiotic stress is shaped by the environment that is confronting the plant. Consequently, changes in environmental conditions can alter the molecular, biochemical, and physiological responses in plants.

China is the largest producer of peanut (17.99 million metric tons) followed by India (6.70 million metric tons) and the USA (2.79 million metric tons); however, the USA produces the highest yields (4.27 metric tons per ha), followed by China (3.79 metric tons per ha) and India (1.12 metric tons per ha) [30]. Peanut is an important cash crop worldwide, producing India’s third-most consumed edible oil. Various abiotic stresses are the key reason for reduced yields in India [31].

Cluster computing and yield simulation suggest that climate change will reduce peanut production by 2.3–33.7% in India [32]. In this study, different abiotic stress combinations were used to simulate possible future climatic conditions and their effects on peanut plants. This study investigated the effect of individual and combined abiotic stresses on the peanut’s physio-biochemical and metabolomic responses, elucidating their effect on the complex metabolic networks and pathways and metabolite accumulation. In addition, multivariate correlation analysis of the physio-biochemical and metabolomic parameters was used to understand the molecular mechanism of abiotic stress responses in peanut plants.

ROS are an integral part of plant sensing and signaling [33,34]. Plants in unstressed environments maintain a delicate balance between ROS production and scavenging, which are involved in cellular signaling. Exposure to abiotic stress causes excess ROS production in plant cells, damaging cellular membranes [35]. DAB (brown precipitate) and NBT (blue precipitate) histochemical staining produce visible staining in leaf explants by reacting with accumulated peroxide and free oxygen radicals, respectively [36]. DAB staining of peanut leaves suggested that combined stresses such as S-H, S-D, D-H, and S-D-H significantly induced higher peroxide radical formation than the individual stresses (Figure 4). Similarly, NBT staining showed that free oxygen radicals accumulated significantly under S-D, S-C, D-H, S-D-H, and S-D-C stress compared to the control and individual stresses (Figure 4). Thus, the combined stresses accumulated more ROS in peanut plants than the individual stresses. Abiotic stresses cause lipid peroxidation of membranes leading to cellular membrane damage and leakage [31]. In *Sesamum indicum* cv. Orhangazi, salt stress significantly increased the lipid peroxidation [37]. In contrast, lipid peroxidation did not significantly change in the individual stress treatments. Interestingly, lipid peroxidation increased in peanut under S-H and S-D-H stress, suggesting the presence of strong antioxidant machinery.

The antioxidative enzyme system of plants scavenges excess ROS and restores homeostasis. A study on peanut plants reported that CAT activity in the leaf tissue decreased by up to 52% under drought stress compared to the control [22]. Similarly, in our study, the CAT activity decreased by 72% under drought stress, more so when combined with salt (S-D, 84%), heat (D-H, 85%), or salt and heat (S-D-H, 94%) stress (Figure 3). Similar results were reported for *Hedychium* plants, where combined drought and heat stress decreased the CAT activity in leaf samples [11]. In contrast, combined cold and drought (D-C, 56%) and salt, drought, and cold (S-D-C, 48%) stress significantly increased CAT activity (Figure 3). Under salt stress, SOD and APX activities decreased by 50–70% in peanut plants [38]. In contrast, the SOD activity increased, and APX activity remained stable under salt stress in the present study compared to the control. The different SOD and APX responses may be related to differences in the genotype and stress duration; for example, 48 h stress with GG20 cultivar in this study compared to 96 h stress with Luhua14 cultivar [38]. The APX activity almost doubled under S-C and S-D-C stress. Glutathione reductase (GR) activity increased significantly in green spurge (*Euphorbia esula*) under individual drought and cold stresses compared to the control [39]. In our study, the GR activity increased under all individual and combined stresses, but more so for the combined stresses that included salt (S-C, S-H, S-D-H; Figure 3).

Plant biochemical constituents such as amino acids, polyphenol, sugars, and starch play important roles in osmotic homeostasis, sensing and signaling, and growth during abiotic stress [40]. The total soluble sugar concentration increased in soybean leaves after long-term drought stress [41]. Similarly, in peanut plants, the sugar and starch concentrations increased under salt, drought, and S-D stress, perhaps acting as osmoprotectants and helping maintain turgor pressure and membrane stability. In Arabidopsis, the accumulation of sugars, amino acids, and polyphenols under drought stress drives ion and osmotic homeostasis [42]. In peanut leaves, FAA concentrations increased in all individual and combined stress treatments except for those that were related to cold stress, indicating that cold stress ameliorates the effect of other concurrent abiotic stresses (Figure 1).

Proline is a particularly important osmoprotectant for plant abiotic stress tolerance [43]. Transgenic tobacco plants that were overexpressing the proline biosynthesis gene pyrroline 5-carboxylate synthetase, had better drought, heat, and drought-heat sequential stress tolerance than control plants, indicating the role of proline accumulation in abiotic stress tolerance [44]. The overexpression of proline-synthesizing genes or the exogenous application of proline enhanced the salt tolerance in plants [45]. In soybean plants, the overexpression of transcription factor GmDREB6 led to proline accumulation and enhanced salinity tolerance [46]. Likewise, proline concentrations, measured by calorimetry, significantly increased under salt, S-H, D-H, S-D, and S-D-H stress, indicating its involvement in plant defense mechanisms against abiotic stress. Especially, significantly more proline accumulated under S-D than individual salt or drought stress, revealing the destructive nature of the combined stress (Figure 1).

In this study, the PCA biplot of metabolites showed that salt stress significantly increased the proline content in peanut plants, confirming its importance for salt stress tolerance (Figure 5). Relatedly, the proline concentration increased in switchgrass by overexpressing proline-synthesizing enzyme genes under multiple individual abiotic stresses such as salt, heat, drought, and cold [47]. Similarly, the PCA biplot shows that proline significantly correlated with D-H stress in this study, suggesting that plants accumulate high proline content under heat and osmotic stress. Amino acid homeostasis plays an essential role in the tolerance mechanism through protein synthesis or degradation [48]. Individual stresses accumulated different amino acids [cold (leucine and valine), drought (asparagine and isoleucine), and heat (glutamic acid and serine)]. Combined stresses that included salt stress (S-D and S-D-H) significantly correlated with isoleucine and tyrosine, whereas D-H significantly correlated with proline, valine, threonine, tryptophan, and phenylalanine.

Polyphenols are a group of plant secondary metabolites with various functions, such as growth and regulation, abiotic stress tolerance, UV-B radiation endurance, and color, sensory, and antioxidant properties [49]. The total polyphenol content and antioxidant activity in green barley increased under combined drought and light stress [50]. Similarly, in peanut plants, drought-related stresses (drought, S-D, D-H, S-D-H) increased the polyphenol content, more so for the combined stresses (Figure 1).

Potato cultivars, Burbank and Unica, had low total chlorophyll concentrations after salt, drought, and combined salt and drought stress [51]. In contrast, salt stress did not affect the chlorophyll content of *Suaeda fruticosa* leaves [52]. Similarly, in peanut plants, only slight (insignificant) changes in pigment content occurred under the individual stresses; however, significant increases occurred under the combined stresses (S-D, D-C, S-D-H, S-D-C). The strong antioxidant machinery and improved osmotic homeostasis in peanut plants might have helped increase the pigment concentration under combined stress. The leaf RWC decreased significantly under drought and D-H stress in two Himalayan plant species, tagar-ganthoda (*Valeriana jatamansi*) and spiked ginger lily (*Hedychium spicatum*) [11]. Likewise, in peanut plants, RWC decreased significantly under drought stress, with additive effects that were observed under combined stresses that included drought (S-D, D-H, and S-D-H). However, cold stress ameliorated the effect of drought, with only a slight decrease in RWC under D-C and S-D-C stress. Electrolyte leakage of tall fescue (*Festuca arundinacea*) increased significantly under individual salt, drought, and heat stresses [53]. Surprisingly, in peanut, EL only increased in the treatments containing heat stress (heat, S-H, D-H, and S-D-H), which may be due to the high membrane stability that was observed in all the heat-treated plants, preventing EL from cells (Figure 2).

Plants change their metabolome to acclimatize to their surrounding environment. Small changes in environmental parameters can trigger changes in metabolic pathways, leading to the synthesis, accumulation, or degradation of different metabolites in cells. Combined stresses alter the metabolome of plants more than individual stresses. Analysis of the effect of combined stress on the peanut metabolome using PCA biplots, pathway enrichment, heatmaps, PLS-DA, and correlation analysis offered valuable insights into abiotic stress tolerance mechanisms.

The stress treatments significantly altered the TCA and urea cycles and their associated amino acid biosynthesis pathway intermediates. The exogenous application of citric acid (TCA intermediate) increased abiotic stress tolerance by improving ROS homeostasis, photosynthetic rates, and osmoregulation [54]. In our study, citric acid concentration decreased in all the stress treatments (individual and combined) except heat, whereas cis-aconitate concentration increased in most heat-related stresses. These results indicate that citric acid was not formed from pyruvate or rapidly converted to cis-aconitate, the next intermediate in the TCA cycle. Increasing the pyruvate concentration increased valine, leucine, and isoleucine biosynthesis as the concentration of these amino acids increased under abiotic stress (Figure 6). Increased cis-aconitate leads to increased GABA or 4-aminobutanoate concentrations, increasing the production of glutamic acid, glycine, and serine.

Recently, the role of urea cycle intermediates such as ornithine, aspartate, arginine, and citrulline was reported in plant abiotic stress tolerance mechanisms [55,56,57,58]. Engineered Arabidopsis overproducing ornithine showed enhanced tolerance to salt and drought stress, which may be due to the increased ornithine producing arginine and aspartate, intermediates of proline biosynthesis [56]. In contrast, the urea concentration decreased significantly in peanut plants under all abiotic stress treatments except for drought and S-D, indicating reduced ornithine co-production. An Arabidopsis mutant of arginine synthase had lower arginine concentrations leading to enhanced byproducts, such as polyamines, NO, and citrulline, involved in abiotic stress tolerance [57]. In peanut plants, the arginine precursor aspartate decreased under most abiotic stresses, increasing the production of asparagine, phenylalanine, tyrosine, alanine, lysine, and proline.

Abiotic stresses significantly affected the plant metabolome by altering metabolic pathways. Pathway enrichment analysis identified the most affected metabolic pathways in peanut plants under individual and combined abiotic stresses. Salt, drought, and salt-drought stress differentially accumulated free amino acids in mangrove (*Avicennia marina*) [59]. Likewise, individual and combined abiotic stresses significantly affected amino acids, especially those in the valine, leucine, and isoleucine biosynthesis pathways. Transcriptome analysis of Chinese cabbage (*Brassica rapa*) under drought stress showed differential expression of transcription factors and acclimation response for glucosinolate metabolism [60]. Similarly, individual and combined stresses significantly affected glucosinolate metabolism in peanut plants. Galactose metabolism led to the production of ascorbic acid, a strong antioxidant during abiotic stress. The manipulation of the galactose metabolism pathway and the overproduction of ascorbic acid enhanced the abiotic stress tolerance in rice [61]. Peanut plants under abiotic stress also had an altered galactose metabolism pathway. Similarly, the sugar metabolism pathway changes to provide abiotic stress tolerance [62]. In peanut plants, the pathways related to sugar metabolism, including pentose and glucuronate interconversion, C5 branched dibasic acid metabolism, and pantothenate and Co-A biosynthesis pathways significantly changed to cope with abiotic stress. Drought stress enhanced indole alkaloid biosynthesis in the medicinally important plant periwinkle (*Catharanthus roseus* var. *rosea*) [63]. Similarly, indole alkaloid biosynthesis increased in peanut plants, enhancing antioxidant activities during abiotic stress.

## 4. Materials and Methods

### 4.1. Plant Material and Stress Conditions

Healthy and mature peanut seeds of a widely used local (Gujarat, India) cultivar (GG-20) were washed with 0.1% (*v*/*v*) Tween-20 solution followed by distilled water. The seeds were surface sterilized with 70% (*v*/*v*) EtOH for 1 min and 0.1% (*w*/*v*) HgCl_2_ for 8 min [64,65]. The seeds were washed five to six times with sterile water and germinated on Petri dishes containing sterile wet filter paper in the dark in a growth room. The seven-day-old germinated seedlings were transferred to a hydroponic culture system [1/2 strength Murashige and Skoog (MS) salts, pH 5.8, with 8/16 h dark/light cycle at 25 °C] for one month, with the culture medium replaced every five days. The plants were subjected to different abiotic stresses, individually or combined, for 48 h (Supplementary Table S2) before harvesting the third and fourth tetra foliate from the top of plants. The leaves were stored immediately in liquid nitrogen for further experiments.

### 4.2. Relative Water Content, Electrolyte Leakage, and Membrane Stability Index

Leaf disks were cut from the stressed and control plants and incubated and gently shaken overnight in deionized water before measuring the fresh weight (FW), turgid weight (after 24 h incubation), and the dry weight (after drying at 65 °C constant temperature). The percent relative water content (RWC, %) was calculated as follows:

RWC (%) = 100 × [(Fresh weight − Dry weight)**/**(Turgid weight − Dry weight)]

The fresh leaf disks were used to measure the membrane stability index (MSI). The first set (L1) of leaves was stored at 40 °C for 30 min and the second set (L2) was stored at 100 °C for 10 min and cooled to room temperature. The electrical conductivity (EC) of both sets was measured with a conductivity meter (SevenEasy, Mettler Toledo, Greifensee, Switzerland). MSI was calculated as 100 × [1 − L1/L2].

For the electrolyte leakage (EL) measurement, the fresh leaf samples were thoroughly rinsed with sterile distilled water to remove surface attached electrolytes. The samples were stored in sterile water in sealed tubes and incubated on a rotating shaker overnight. The EC was determined (Lt) with a conductivity meter the next day. The leaf samples from each treatment were then autoclaved at 120 °C for 15 min, cooled to room temperature, and the EC (L0) was re-measured [66]. The percentage EL was determined as EL (%) = 100 × Lt/L0.

### 4.3. Estimation of Photosynthetic Pigments and Na^+^/K^+^ Ratio

Fresh leaf from the stressed and control plants was used to estimate the various photosynthetic pigments. Leaf tissue (20 mg) was extracted in 100% N, N-dimethylformamide (DMF) at 4 °C to estimate Chl *a*, Chl *b*, total Chl (*a* + *b*), and carotenoid contents by recording the absorbance at 664.5, 664, 647, and 461 nm, respectively [67,68].

For the Na^+^ and K^+^ contents, the dry leaf samples (100 mg) were digested in perchloric acid and nitric acid (9:4 *v*/*v* ratio), evaporated to dryness on a hotplate, and the residue was dissolved in 50 mL deionized water. The solution was filtered through 0.22 µm syringe filter before measuring the Na^+^ and K^+^ contents with an inductively coupled plasma atomic absorption spectrometer (Optima 2000DV, Perkin Elmer, Waltham, MA, USA) [69].

### 4.4. In Vivo Localization of H_2_O_2_ and O_2_^−^

The hydrogen peroxide and O_2_^−^ radical accumulation were detected in vivo [33]. The leaf samples were dipped in nitro-blue tetrazolium (NBT) solution (1 mg mL^−1^ NBT in 10 mM phosphate buffer; pH 7.8) at room temperature for 4 h in the dark. The presence of H_2_O_2_ was detected by dipping the leaf samples in 3,3-diaminobenzidine (DAB) solution (1 mg mL^−1^ DAB, 0.1% Triton X-100, 10 mM Na_2_HPO_4_; pH 3.8) at room temperature for 6 h in the dark before exposing them to the light until brown spots appeared (evidence of H_2_O_2_ accumulation). The chlorophyll content was bleached with ethanol, and photographs were taken of the leaf samples.

### 4.5. Estimation of Free Amino Acids, Polyphenols, and Sugar Contents

The leaf tissue (250 mg) was extracted in 80% ethanol and centrifuged at 10,000× *g* for 10 min. The pellet was used for starch content estimation, with the supernatant left after evaporation dissolved in deionized water to quantify the free amino acids, polyphenol, total soluble sugars, and reducing sugars. Glycine, catechol, and glucose were used as the reference standard for free amino acid, polyphenol, and sugar content estimation, respectively. The total free amino acid contents in the leaf tissues were determined using ninhydrin reagent [70]. The polyphenol contents in the leaves were determined by Folin-Ciocalteau’s reagent [71]. The reducing sugars were computed using a colorimetric test and DNS method [72]. The total soluble sugars and starch (after digestion of pellet with 52% perchloric acid for 2 h) were estimated using anthrone-sulfuric acid [73].

### 4.6. Estimation of Proline Content, Lipid Peroxidation, and Antioxidant Enzyme Activity

About 200 mg leaf samples were crushed in liquid nitrogen and extracted in 3% aqueous sulfosalicylic acid solution for proline content estimation. L-proline (Sigma, St. Louis, MO, USA) was used for standard curve preparation, and a ninhydrin reagent-based method was used for free proline estimation [74]. Lipid peroxidation was calculated by estimating the malondialdehyde (MDA) content that was extracted in 0.1% trichloroaceatic acid solution followed by derivatization with 0.65% thiobarbituric acid in 20% trichloroacetic acid [75].

A ~1 g leaf sample was homogenized in liquid nitrogen before extracting total protein in 10 mL of ice-cold protein extraction buffer (50 mM phosphate buffer, pH 7.0; 1 mM EDTA, pH 8.0; 0.05% *w*/*v* Triton x-100 and 5% *w*/*v* PVPP). The total protein concentration in the extract was determined using the Bradford method; the same protein extract was used to determine the antioxidant enzyme activities, including catalase (CAT), superoxide dismutase (SOD), glutathione reductase (GR), and ascorbate peroxidase (APX).

The SOD activity was measured by monitoring the photo-reduction of NBT [76], with one unit of SOD activity considered to inhibit 50% photo-reduction of NBT. The CAT activity was measured following Miyagawa et al. [77]; briefly, 20 µg protein was added to the reaction mixture (10 mM H_2_O_2_, 50 mM potassium phosphate buffer, pH 7.0), with the decrease in absorbance at 240 nm recorded for 2 min. The APX activity was measured following Nakano and Asada [78]; briefly, H_2_O_2_ was added to initiate the reaction in the reaction mixture (0.5 mM ascorbic acid, 0.1 mM H_2_O_2_, 50 µg of protein enzyme extract, and 50 mM potassium phosphate), with the decrease in absorbance at 290 nm recorded for 1 min. The GR activity was determined by observing the rate of reduction of DTNB; briefly, NADPH was added to the reaction mixture (1 mM EDTA, 1 mM oxidized glutathione (GSSG), 0.75 mM DTNB, 0.1 mM NADPH, 40 µg protein, and 100 mM potassium phosphate, pH 7.5), with the increase in absorbance at 412 nm recorded for 3 min [79].

### 4.7. Transcript Profiling of Antioxidant Enzymes Encoding Genes

Total RNA was isolated from the control and stressed plant samples using PureLink Plant RNA Reagent (Invitrogen, Waltham, MA, USA) and quantified with a Nanodrop spectrophotometer (NanoDrop, Wilmington, DE, USA). The cDNA was prepared using a Qiagen first-strand cDNA synthesis kit. Quantitative real-time PCR (qRT-PCR) reactions were carried out with gene-specific primers, with actin used as the internal reference gene (Supplementary Table S3). The qRT-PCR data were analyzed using the comparative C_T_ method, with the relative fold-gene expression (2^−ΔΔCT^) of APX, SOD, and CAT genes determined after normalizing their expression with internal control actin C_T_-values [80].

### 4.8. Total Antioxidant, Scavenging, and Reducing Activities

The antioxidant potency of the plant extract was determined by estimating the inhibition of free cationic radicals of ABTS (2,2-azino-bis(3-ethylbenzothiazoline-6-sulphonic acid)) compared to Trolox (6-hydroxy-2,5,7,8-tetramethylchroman-2-carboxylic acid) used as the standard. ABTS free radicals were generated by mixing ABTS diammonium salt (7 mM) and potassium persulfate (2.45 mM), followed by incubation at ambient temperature for 12–16 h in the dark. The absorbance was set to ~0.70 ± 0.02 by diluting the reaction mixture with water at 734 nm. Different concentrations (100–500 µg mL^−1^) of plant extract were mixed with 1 mL ABTS ions and incubated for 90 min before recording absorbance at 734 nm; the total antioxidant activity was expressed as percentage inhibition [81,82].

The scavenging activity was measured as percent inhibition of DPPH (2,2-diphenyl-1-picrylhydrazyl) free radicals. A DPPH solution containing methanol (0.024% *w*/*v*) was diluted to read 0.98 ± 0.02 absorbance at 517 nm, before mixing about 1 mL with various concentrations (100–500 µg/mL) of plant extract; the percentage radical scavenging activity was compared with the control at 517 nm [83,84,85].

For reducing activity, 1 mL phosphate buffer (0.2 M, pH 6.6) and 1 mL potassium ferricyanide (K_3_Fe(CN)_6_; 10 mg mL^−1^) were mixed with ascorbic acid or different concentrations (100–500 µg mL^−1^) of plant extract and incubated in a 50 °C water bath for 20 min. The reaction was terminated by adding trichloroacetic acid before centrifuging at 7000× *g* for 10 min at room temperature. About 1 mL of the supernatant was blended with freshly prepared 0.2 mL ferric chloride (0.1%, *w*/*v*) and incubated at room temperature for 10 min, before measuring the absorbance at 700 nm [86,87].

### 4.9. Total Phenolic and Flavonoid Contents

The total phenolic content (TPC) and total flavonoid content (TFC) were calculated using standard gallic acid and quercetine that was equivalent per 100 mg of extract, respectively [88]. For TPC, different amounts (100–500 µg mL^−1^) of plant extract were combined with Folin-Coicalteu reagent (0.2 M) and incubated for 5 min, before adding 2 mL sodium carbonate (Na_2_CO_3_; 75 g L^−1^), and reading the absorbance after dark incubation for 90 min. For TFC, different concentrations (100–500 µg mL^−1^) of plant extract were mixed with 0.3 mL sodium nitrite (NaNO_2_, 5%, *w*/*v*) and incubated at ambient temperature for 5 min. The absorbance of the reaction mixture was read at 510 nm after adding 0.3 mL aluminum chloride (AlCl_3_, 10%, *w*/*v*) and 2 mL sodium hydroxide (NaOH, 1 M).

### 4.10. Metabolite Profiling

Metabolite profiling of the plant samples was performed using GC-MS (gas chromatography-mass spectroscopy). The plant leaves (100 mg) were powdered in liquid nitrogen using a mortar and pestle for metabolite extraction using ice-cold methanol [89,90]. The extracted metabolites were derivatized using methoxyamine hydrochloride and MSTFA (N-methyl-N-(trimethylsilyl) trifluoroacetamide). Ribitol/adonitol (6 µg) was used as an internal standard. The GC analysis was performed using 1 µL sample in a flame ionization detection (FID)/capillary column that was equipped with an autosampler (AOC-5000, Shimadzu, Japan). The peaks were identified and verified by comparison with the library of mass spectra (NIST) and quantified using the internal standard.

### 4.11. Statistical Analysis

Each experimental set comprised of data from three biological replicates. The data were expressed as the mean ± SE and analysis of variance (ANOVA) was used to determine significant differences between the treatments using IBM SPSS ver. 27. For multiple means comparison, LSD was used, with *p* < 0.05 considered statistically significant. MetaboAnalyst ver. 5.0 was used for the pathway enrichment, PLS-DA, and correlation analyses.

## 5. Conclusions

The combined abiotic stresses affected the physio-biochemical and metabolic pathways in peanut plants more than the conventionally studied individual stresses, suggesting the need to emphasize stress combinations to validate newly developed peanut cultivars. Moreover, the metabolomics data revealed the differential accumulation of important metabolites (pinitol, malic acid, and xylopyranose) under abiotic stress conditions. In the future, key genes of these biosynthesis pathways should be functionally validated under combined stress conditions to confirm their precise role in the combined stress tolerance mechanism. Superior peanut cultivars with abiotic stress tolerance can be developed using identified molecular phenotypes and traditional breeding or genetic engineering. In conclusion, this study provides a new perspective for crop improvement programs under the changing climate. Future studies could identify the stressor markers that are unique to the stress and independent of the cultivar to infer a model to detect resistant phenotypes among different varieties.

## Figures and Tables

**Table 1:** Comprehensive metabolite profiling of peanut plants under different abiotic stresses.

Metabolites	Control	Salt	Drought	Heat	Cold	S-H	S-C	S-D	D-H	D-C	S-D-H	S-D-C
**Amino acids**												
Valine	0.185 ± 0.004	0.411 ± 0.179			4.360 ± 0.097	2.543 ± 0.001	0.467 ± 0.224		8.035 ± 1.411	0.495 ± 0.046		0.736 ± 0.058
Proline	0.641 ± 0.084	8.815 ± 4.17		0.667 ± 0.090	1.159 ± 0.138	0.721 ± 0.005	0.427 ± 0.135		10.043 ± 2.103	0.798 ± 0.160		0.667 ± 0.074
Glycine	0.048 ± 0.011	0.515 ± 0.211	0.379 ± 0.044		0.155 ± 0.024	0.052 ± 0.002	0.031 ± 0.010	0.252 ± 0.282	0.491 ± 0.148	0.030 ± 0.004		0.384 ± 0.034
Serine	2.269 ± 0.093	3.674 ± 2.041	2.203 ± 0.180	3.548 ± 0.185	1.295 ± 0.113		2.688 ± 0.891	0.927 ± 0.182	2.774 ± 0.677	2.397 ± 0.365	0.855 ± 0.112	3.250 ± 0.319
Threonine	0.808 ± 0.037	0.734 ± 0.367	2.827 ± 0.080	0.865 ± 0.036	5.222 ± 0.153	4.466 ± 2.033	0.818 ± 0.272	2.179 ± 0.652	9.853 ± 1.386	1.016 ± 0.060	2.623 ± 0.174	1.263 ± 0.039
Alanine			0.204 ± 0.030	0.092 ± 0.005	0.082 ± 0.005	0.078 ± 0.003	0.054 ± 0.025	0.149 ± 0.030	0.340 ± 0.137	0.052 ± 0.010	0.125 ± 0.004	0.244 ± 0.037
Aspartic acid	7.479 ± 0.344		5.438 ± 0.153	14.661 ± 1.653		2.475 ± 0.026	5.278 ± 1.892			7.003 ± 0.581	0.800 ± 0.034	9.445 ± 0.807
Glutamic acid	14.667 ± 0.397	4.559 ± 2.548	6.392 ± 0.711	20.089 ± 0.955	11.722 ± 0.818	2.846 ± 0.016	11.715 ± 3.699	4.839 ± 1.216	2.712 ± 0.379	10.352 ± 0.562	1.029 ± 0.060	13.590 ± 1.004
Phenylalanine		0.217 ± 0.107	3.710 ± 0.194	0.434 ± 0.014	8.9769 ± 0.521	5.161 ± 2.207	0.320 ± 0.105	3.368 ± 1.069	13.840 ± 1.511	0.363 ± 0.005	3.095 ± 0.143	0.551 ± 0.012
Asparagine	2.240 ± 0.209		7.079 ± 0.916	1.445 ± 0.090		20.593 ± 8.386	3.092 ± 1.118	4.588 ± 0.632	79.016 ± 7.795	2.109 ± 0.162	12.351 ± 1.849	4.608 ± 1.038
Glutamine	0.989 ± 0.070	0.523 ± 0.194	3.434 ± 0.022	1.132 ± 0.340	2.902 ± 0.119	4.800 ± 0.207	1.459 ± 0.556					
Hydroxyproline	8.006 ± 0.520	1.556 ± 0.841	3.695 ± 0.137	6.059 ± 1.318	2.286 ± 0.133	5.873 ± 0.003	4.254 ± 1.399	1.871 ± 0.291	1.407 ± 0.010	5.637 ± 0.986	0.837 ± 0.058	8.749 ± 0.925
Lysine	4.880 ± 0.029		2.316 ± 0.282	0.063 ± 0.0064	2.136 ± 0.352	4.880 ± 0.029	0.408 ± 0.149	1.692 ± 0.375	4.067 ± 0.622	0.537 ± 0.060	2.077 ± 0.189	0.920 ± 0.062
Tyrosine			0.716 ± 0.038	0.339 ± 0.087	0.473 ± 0.058	0.668 ± 0.310	0.498 ± 0.184	1.010 ± 0.319	0.798 ± 0.191	0.575 ± 0.053	0.666 ± 0.036	0.957 ± 0.044
**Dicarboxylic acids**												
Butanedioic acid	0.642 ± 0.077	0.406 ± 0.077		0.551 ± 0.184	1.159 ± 0.092	1.204 ± 0.382	0.480 ± 0.151	1.235 ± 0.655	1.793 ± 0.384	0.742 ± 0.108		1.185 ± 0.093
2-Butenedioic acid	0.377 ± 0.025	0.243 ± 0.102	0.518 ± 0.074	0.252 ± 0.034	0.207 ± 0.016	0.194 ± 0.067	0.262 ± 0.089	0.248 ± 0.052	0.467 ± 0.142	0.310 ± 0.051	0.317 ± 0.18	0.482 ± 0.034
Malic acid	17.297 ± 1.652	19.351 ± 8.646	11.940 ± 1.687	30.363 ± 3.380	19.820 ± 6.580	6.895 ± 2.340	10.003 ± 3.138	6.049 ± 1.017	36.197 ± 6.523	10.609 ± 0.708	4.246 ± 0.372	15.174 ± 0.340
4-Aminobutanoic acid	0.533 ± 0.071	0.204 ± 0.090	1.746 ± 0.302			0.263 ± 0.003	0.702 ± 0.253	1.133 ± 0.365	1.887 ± 0.467	0.834 ± 0.089	1.414 ± 0.084	
Pentanedioic acid	0.579 ± 0.023	0.210 ± 0.119	0.505 ± 0.055	0.348 ± 0.034	0.204 ± 0.031	0.740 ± 0.241	0.152 ± 0.053	0.362 ± 0.105	0.455 ± 0.136	0.194 ± 0.036	0.285 ± 0.026	0.231 ± 0.012
Tartaric acid	2.049 ± 0.312	0.824 ± 0.350	2.752 ± 0.853	3.225 ± 0.695		0.961 ± 0.290	0.629 ± 0.212		2.155 ± 0.716	1.284 ± 0.078	0.573 ± 0.099	
Methylsuccinic acid	0.116 ± 0.034		0.068 ± 0.006			0.056 ± 0.020		0.017 ± 0.000			0.0577 ± 0.000	0.167 ± 0.043
**Fatty acids**												
Glyceric acid		0.215 ± 0.090		0.348 ± 0.368		0.050 ± 0.003	0.206 ± 0.068	0.062 ± 0.018	0.191 ± 0.085	0.254 ± 0.040	0.218 ± 0.008	0.279 ± 0.003
Palmitic acid	1.718 ± 0.333	0.217 ± 0.143	0.446 ± 0.010	0.609 ± 0.1534	0.353 ± 0.078	0.506 ± 0.169	0.602 ± 0.159	0.273 ± 0.062	-	0.673 ± 0.105	0.194 ± 0.005	0.575 ± 0.026
**Alcoholic groups**												
Ethanolamine	1.078 ± 0.03	1.078 ± 0.456	1.092 ± 0.051	2.042 ± 0.040	1.219 ± 0.143	0.622 ± 0.003	1.035 ± 0.324	0.672 ± 0.169	1.207 ± 0.189	1.099 ± 0.092		1.271 ± 0.137
Silanol	12.323 ± 1.559	5.611 ± 3.181	7.268 ± 1.070	22.939 ± 3.093	14.740 ± 2.532	4.794 ± 1.577	5.725 ± 1.897	3.047 ± 0.689	7.872 ± 2.511	7.079 ± 0.690		9.264 ± 0.595
**Aromatics**												
Pyrazine	0.275 ± 0.050	0.129 ± 0.015		0.137 ± 0.137	5.258 ± 0.527			0.662 ± 0.001	17.777 ± 1.049	0.553 ± 0.082		0.504 ± 0.070
Shikimic acid	0.103 ± 0.028	0.370 ± 0.157	0.585 ± 0.132	0.891 ± 0.312	0.607 ± 0.170	0.520 ± 0.199	0.517 ± 0.196	0.335 ± 0.021	0.286 ± 0.109	0.650 ± 0.108	0.127 ± 0.040	1.421 ± 0.111
Quininic acid		7.233 ± 2.744	5.944 ± 1.343		15.007 ± 5.811	2.978 ± 1.111	8.165 ± 2.673	5.274 ± 0.568	13.415 ± 5.334	7.852 ± 1.054	3.479 ± 0.403	16.217 ± 2.093
Pinitol	34.372 ± 2.76	59.312 ± 18.71	35.060 ± 2.765	71.672 ± 4.223	99.445 ± 4.692	23.775 ± 8.061	39.056 ± 11.678	21.731 ± 4.947	148.20 ± 21.20	39.972 ± 1.590	17.050 ± 0.968	45.476 ± 5.153
Caffeic acid	0.233 ± 0.143		1.581 ± 0.119		0.645 ± 0.167	0.690 ± 0.279	0.715 ± 0.278	1.350 ± 0.325	1.486 ± 0.341	0.634 ± 0.284	2.525 ± 0.096	3.101 ± 0.410
**Sugars**												
Melibiose	0.723 ± 0.037			0.689 ± 0.1652		0.604 ± 0.019	0.537 ± 0.188	0.542 ± 0.191	0.269 ± 0.087			0.586 ± 0.030
Mannobiose	0.802 ± 0.049		0.672 ± 0.033	0.518 ± 0.0476		0.186 ± 0.073	0.439 ± 0.169	0.317 ± 0.091		0.777 ± 0.021		0.770 ± 0.052
Sucrose	63.910 ± 6.050	73.783 ± 16.69	42.000 ± 2.939	66.401 ± 4.275	3.343 ± 0.846	1.513 ± 0.595	44.004 ± 13.313	35.012 ± 11.70	6.887 ± 0.633	39.878 ± 1.824	1.518 ± 0.069	54.621 ± 3.886
**Miscellaneous**												
Propanedioic acid	0.070 ± 0.001		0.415 ± 0.010	0.586 ± 0.214	0.517 ± 0.026	0.303 ± 0.106	0.040 ± 0.013	0.215 ± 0.052	0.556 ± 0.044	0.064 ± 0.007	0.395 ± 0.16	0.165 ± 0.014
Urea	0.535 ± 0.108		0.592 ± 0.006		0.093 ± 0.001	0.238 ± 0.097	0.123 ± 0.038	0.940 ± 0.266	0.295 ± 0.063	0.097 ± 0.035	0.279 ± 0.025	0.289 ± 0.042
Citric acid	13.080 ± 0.461	1.899 ± 0.993	3.765 ± 1.084	31.629 ± 10.214	12.569 ± 1.600	3.897 ± 1.171	4.889 ± 1.752	2.409 ± 0.633	2.647 ± 0.762	5.538 ± 0.509	0.711 ± 0.123	6.835 ± 0.042

## Data Availability

Not applicable.

## Supplementary Materials

The following are available online at https://www.mdpi.com/article/10.3390/ijms23020660/s1.
