# Supplementary material for: Differential Physio-Biochemical and Metabolic Responses of Peanut (Arachis hypogaea L.) under Multiple Abiotic Stress Conditions
Source: Int J Mol Sci. 2022 Jan 8;23(2):660. doi: 10.3390/ijms23020660 (PMC8776106; doi:10.3390/ijms23020660)
Supplement: Supplementary file 1 [file ijms-23-00660-s001.zip › ijms-1531516-supplementary.pdf]

Supplementary data

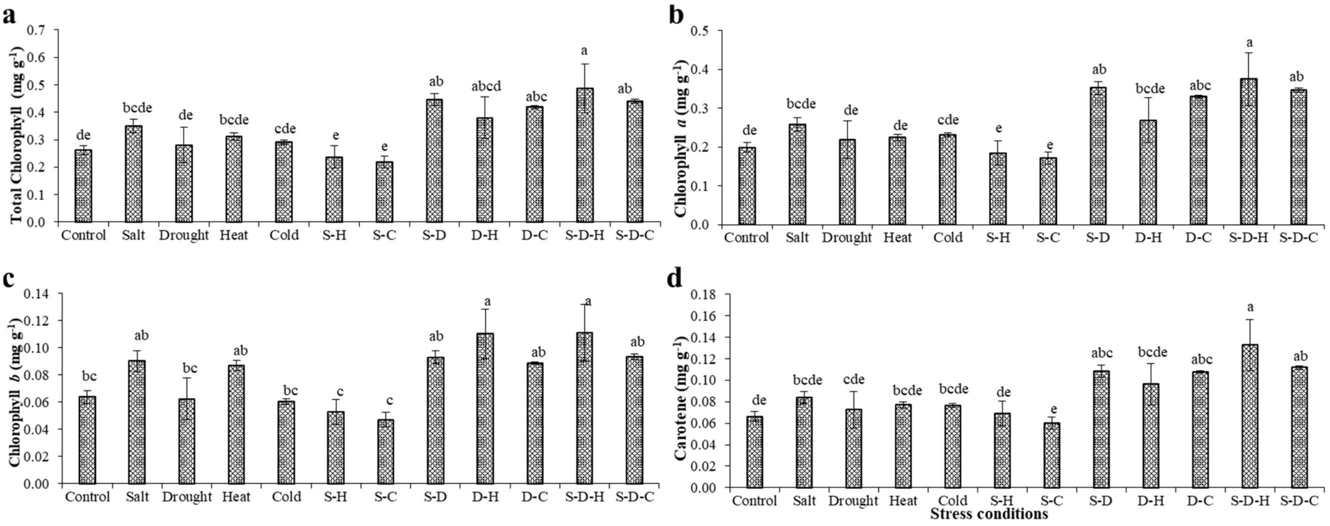

Supplementary Figure S1. Estimation of (a) total chlorophyll (mg g^{-1} FW), (b) chlorophyll *a* (mg g^{-1} FW), (c) chlorophyll *b* (mg g^{-1} FW), and (d) total carotene content (mg g^{-1} FW) in peanut plants treated with different individual and combined stresses (S-D: salinity and drought, S-H: salinity and heat, S-C: salinity and cold, D-H: drought and heat, D-C: drought and cold, S-D-H: salinity, drought, and heat, and S-D-C: salinity, drought, and cold). Data are shown as mean ± SE, and different letters indicate significant differences at $p<0.05$.

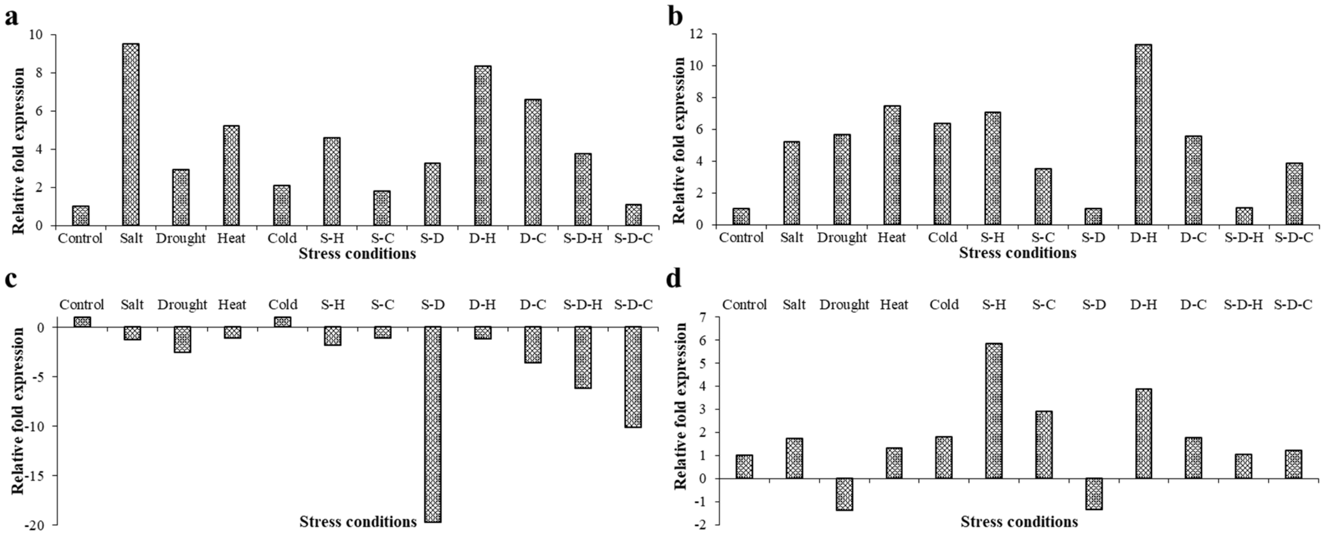

Supplementary Figure S2. Transcript expression analysis of genes encoding antioxidant enzymes. Estimation of relative fold expression of genes encoding for (a) superoxide dismutase (SOD), (b) ascorbate peroxidase (APX), (c) catalase (CAT), and (d) glutathoine reductase (GR) antioxidant enzymes in peanut plants treated with different individual and combined stresses (S-D: salinity and drought, S-H: salinity and heat, S-C: salinity and cold, D-H: drought and heat, D-C: drought and cold, S-D-H: salinity, drought, and heat, and S-D-C: salinity, drought, and cold). Data are normalized with the fold expression of a housekeeping gene (actin).

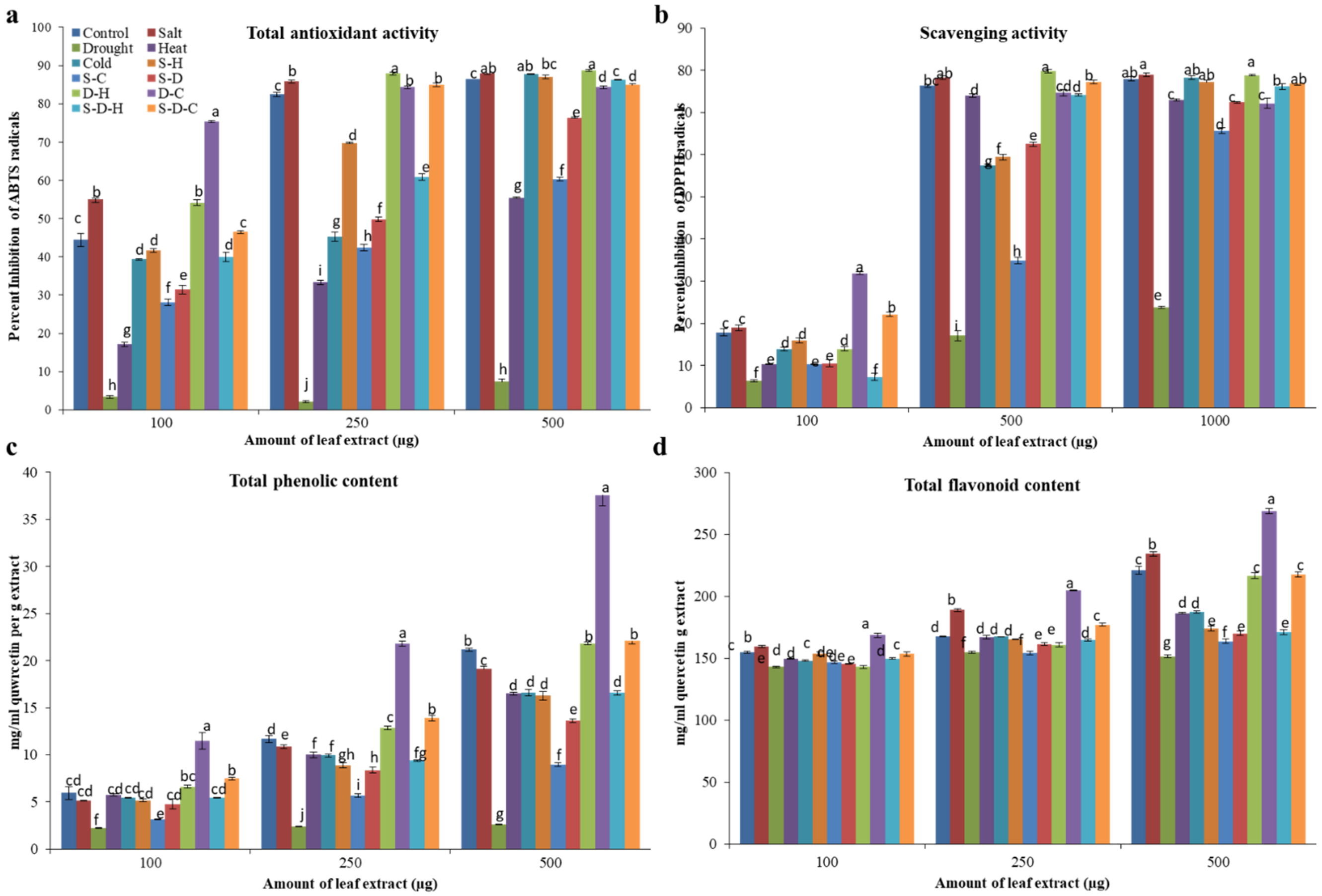

Supplementary Figure S3. Estimation of (a) total antioxidant activity, (b) scavenging activity, (c) total phenolics content, and (d) total flavonoid contents in peanut plants treated with different individual and combined stresses (S-D: salinity and drought, S-H: salinity and heat, S-C: salinity and cold, D-H: drought and heat, D-C: drought and cold, S-D-H: salinity, drought, and heat, and S-D-C: salinity, drought, and cold). Data are shown as mean ± SE, and different letters indicate significant differences at *p*<*0.05*.

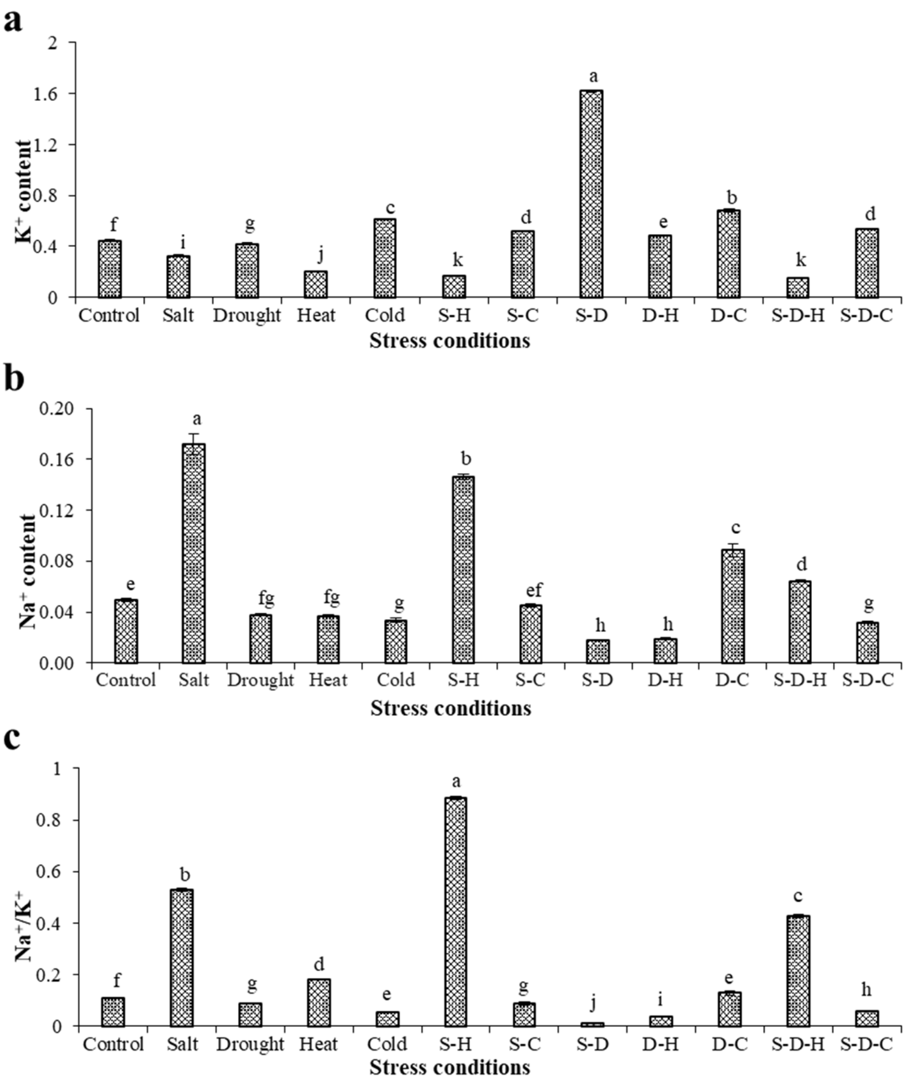

Supplementary Figure S4. Ion content analysis. Estimation of activity of (a) K^+ content (wt%), (b) Na^+ content (wt%), and (c) Na^+/K^+ ratio in peanut plants treated with different individual stresses and combined stresses (S-D: salinity and drought, S-H: salinity and heat, S-C: salinity and cold, D-H: drought and heat, D-C: drought and cold, S-D-H: salinity, drought, and heat, and S-D-C: salinity, drought, and cold). Data are shown as mean ± SE, and different letters indicate significant differences at $p<0.05$.

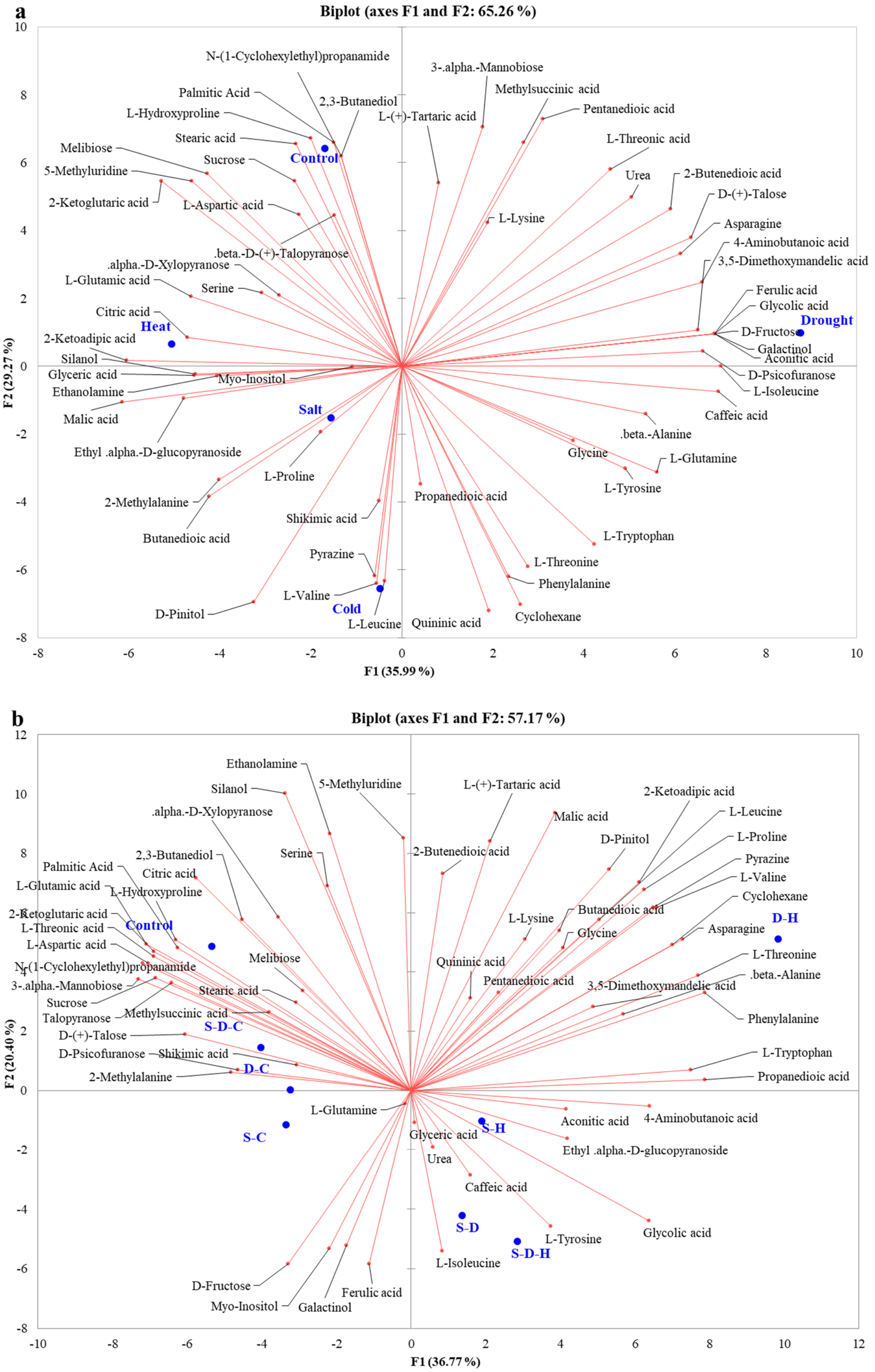

Supplementary Figure S5. Correlation analysis (by PCA biplot) among different metabolites detected in peanut plants treated with different (a) individual and (b) combined stresses (S-D: salinity and drought, S-H: salinity and heat, S-C: salinity and cold, D-H: drought and heat, D-C: drought and cold, S-D-H: salinity, drought, and heat, and S-D-C: salinity, drought, and cold).

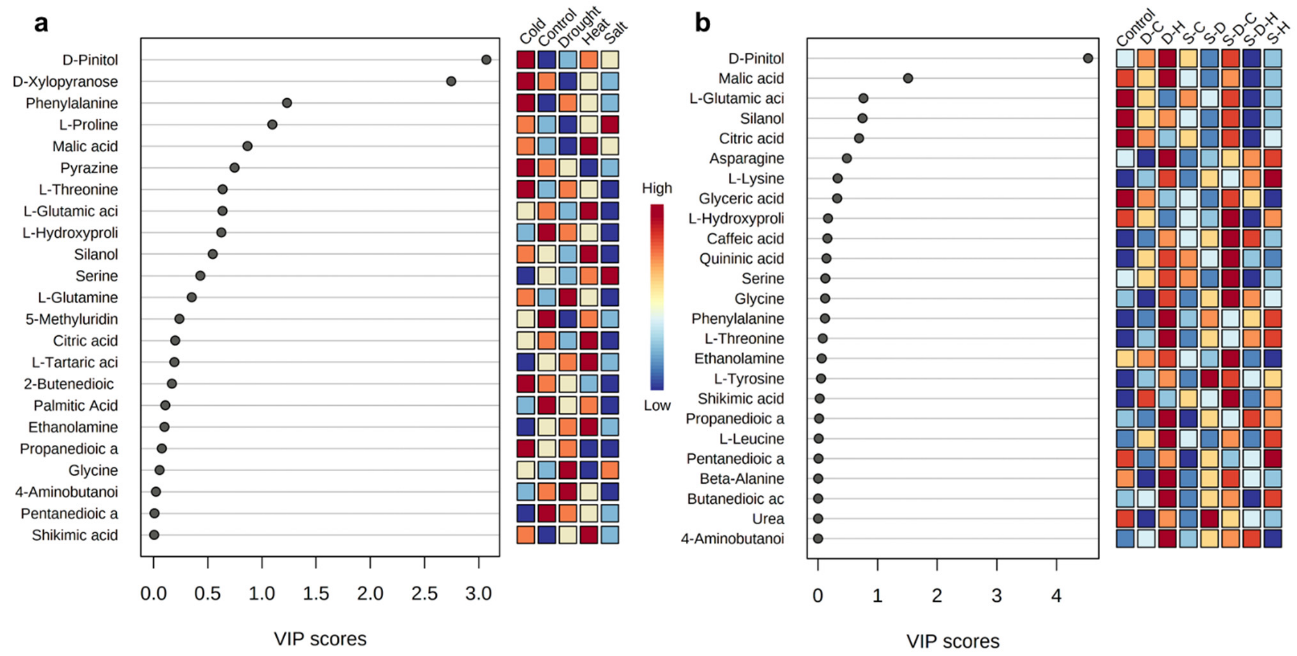

Supplementary Figure S6. Partial least square discriminant analysis (PLSDA) of selected metabolites in peanut plants treated with (a) individual and (b) combined stresses.

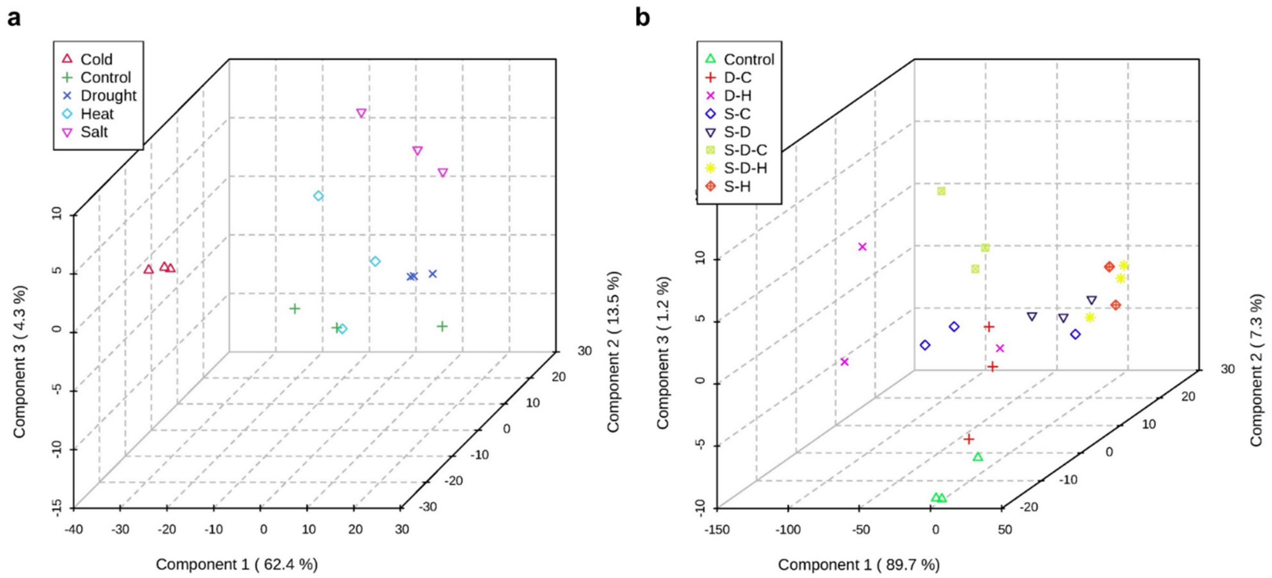

Supplementary Figure S7. 3D score plot between selected principal components for (a) individual and (b) combined stresses.

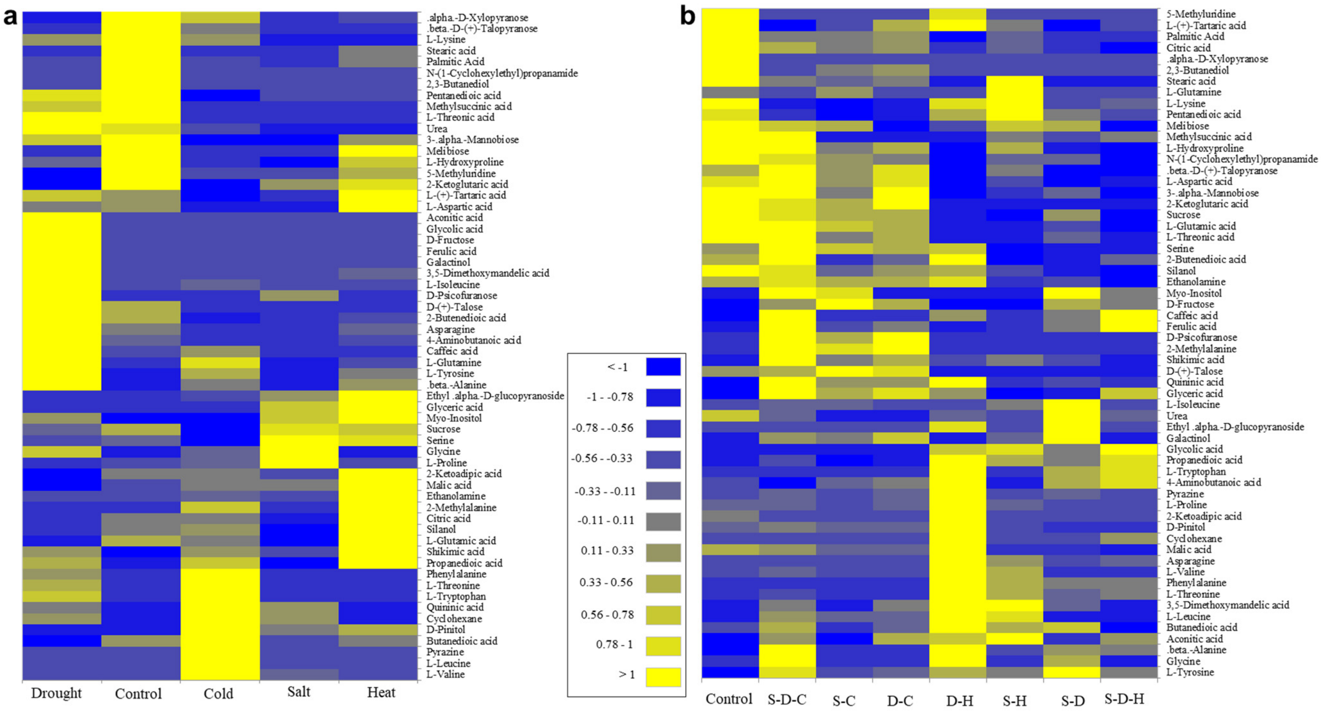

Supplementary Figure S8. Heat map analysis showed differential accumulation of metabolites in peanut plants treated with (a) individual and (b) combined stresses.

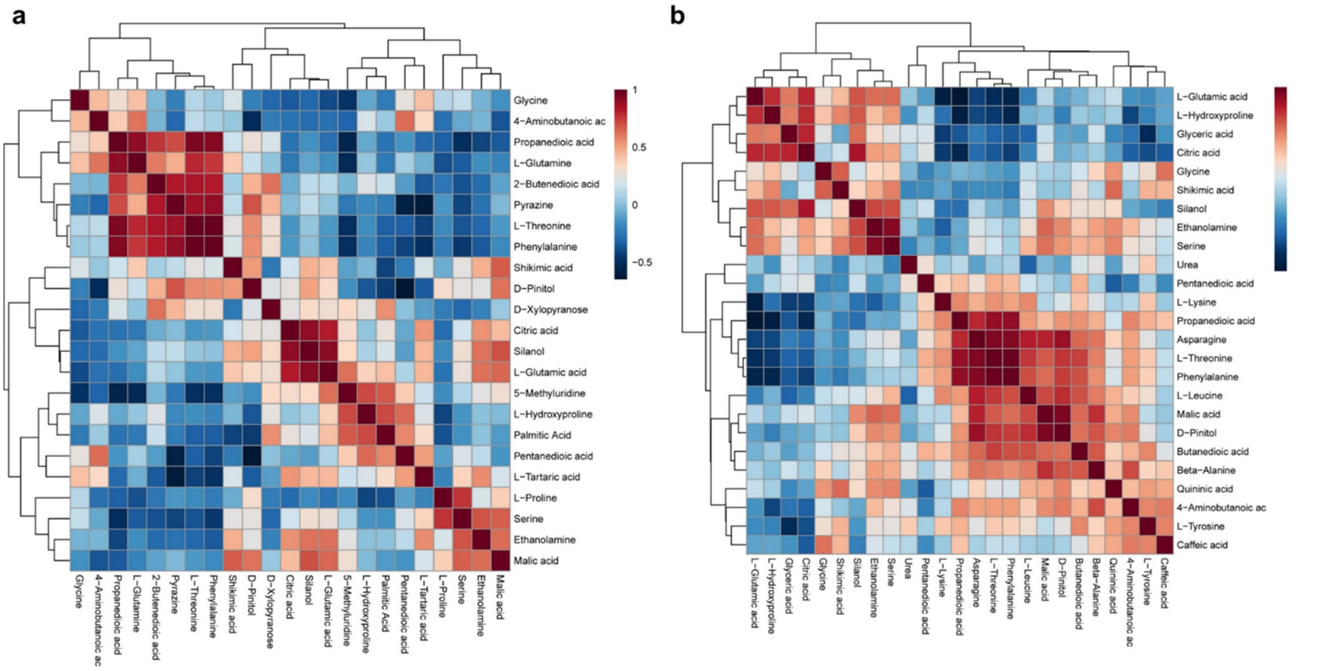

Supplementary Figure S9. Correlation analysis of selected metabolites in peanut treated with (a) individual and (b) combined stresses.

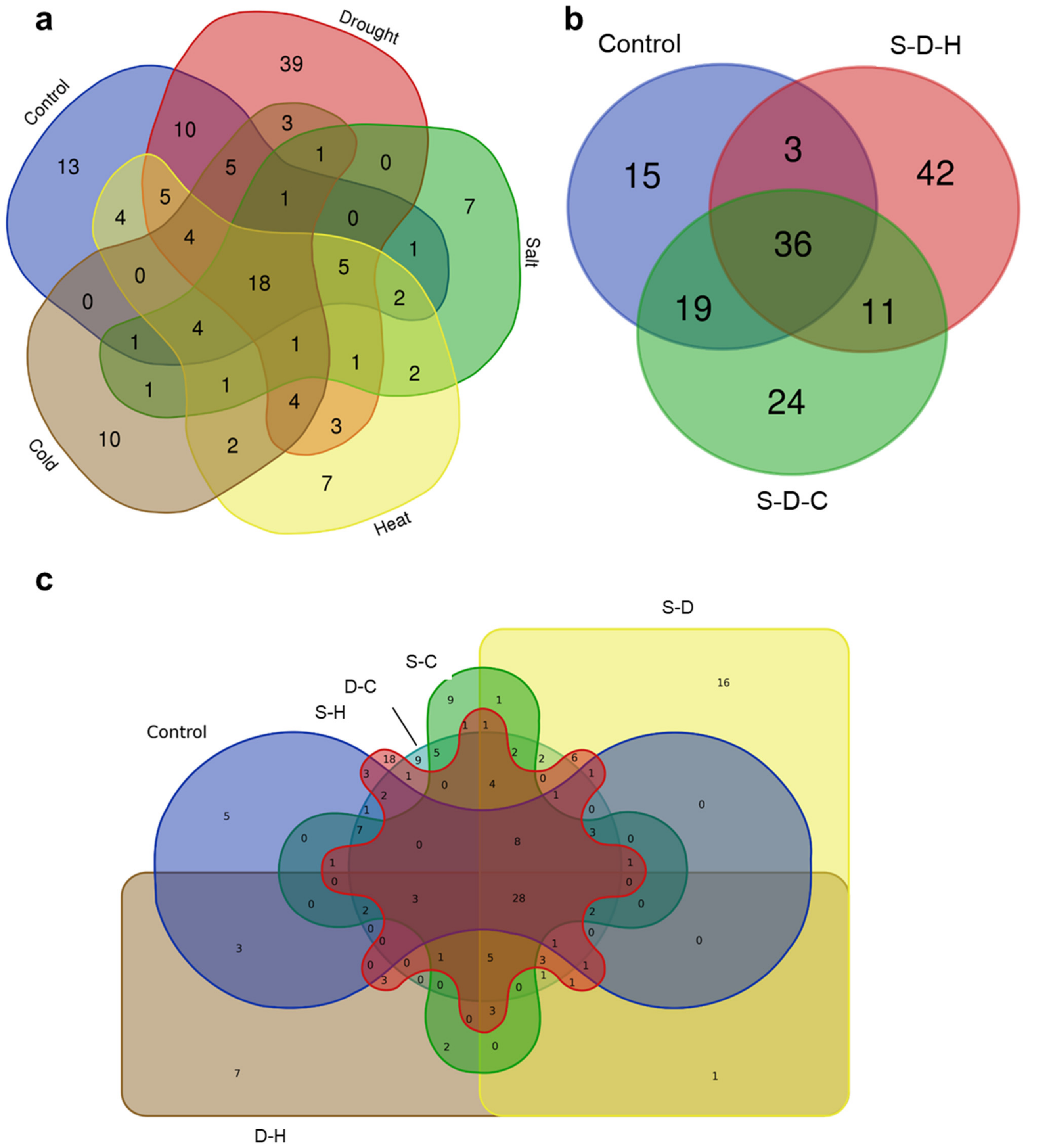

Supplementary Figure S10. Venn diagram of metabolites detected in peanut plants treated with (a) individual stresses, (b) two combined stresses, and (c) three combined stresses.

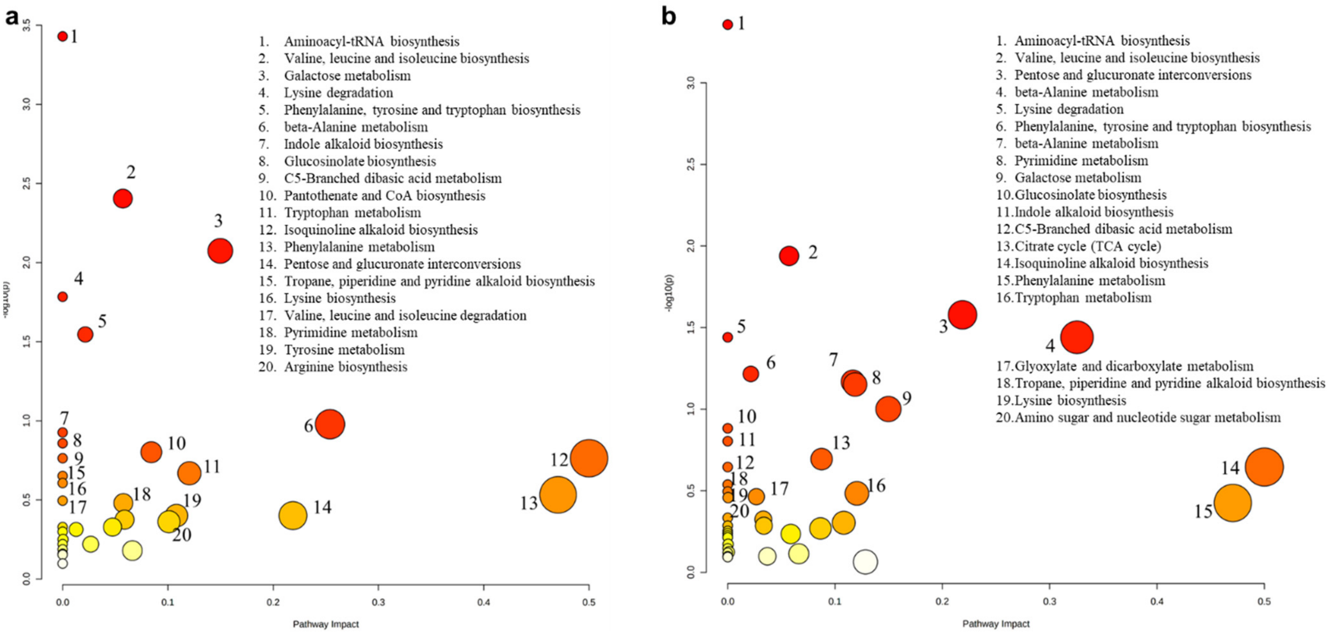

Supplementary Figure S11. Pathway enrichment analysis showed significantly affected metabolic pathways in peanut plants treated with (a) individual and (b) combined stresses.

Table S1. Correlation matrix of different physio-biochemical activities and contents observed in peanut plants treated with different individual and combined stresses.

Correlation matrix (Pearson):

Variables	Proline	ROS	MDA	H_2O_2	ABTS	DPPH	TPC	TFC	CAT	APX	GR	SOD	FAA	PP	Starch	TS	RS	K^+	Na^+	Na^+/K^+	EL	RW	MSI	TC
Proline	**1**	**0.714**	0.114	**0.714**	–0.197	–0.175	–0.132	–0.054	–0.428	–0.122	**0.578**	0.430	0.331	**0.740**	**0.658**	**0.926**	**0.800**	**0.659**	0.072	0.082	0.035	–0.550	0.498	0.485
ROS	**0.714**	**1**	0.498	**1.000**	–0.324	–0.296	0.086	–0.152	–0.565	–0.121	**0.595**	0.576	**0.642**	**0.822**	0.499	**0.613**	**0.579**	0.459	–0.172	0.089	0.427	**–0.680**	**0.632**	**0.626**
MDA	0.114	0.498	**1**	0.498	–0.137	–0.114	–0.068	–0.146	–0.474	0.001	0.470	0.272	0.560	0.256	–0.074	–0.054	–0.090	–0.457	0.357	**0.702**	**0.832**	–0.279	**0.603**	0.249
H2O2	**0.714**	**1.000**	0.498	**1**	–0.324	–0.296	0.086	–0.152	–0.565	–0.121	**0.595**	0.576	**0.642**	**0.822**	0.499	**0.613**	**0.579**	0.459	–0.172	0.089	0.427	**–0.680**	**0.632**	**0.626**
ABTS	–0.197	–0.324	–0.137	–0.324	**1**	**0.989**	–0.572	–0.445	–0.135	–0.361	–0.018	0.396	–0.072	0.119	0.292	0.087	0.337	–0.067	–0.184	–0.166	–0.237	–0.226	0.159	–0.248
DPPH	–0.175	–0.296	–0.114	–0.296	**0.989**	**1**	**–0.660**	–0.535	–0.137	–0.308	0.037	0.413	–0.056	0.140	0.296	0.111	0.351	–0.051	–0.196	–0.153	–0.233	–0.227	0.189	–0.308
TPC	–0.132	0.086	–0.068	0.086	–0.572	**–0.660**	**1**	**0.872**	0.189	0.030	–0.210	–0.419	–0.114	–0.234	–0.264	–0.247	–0.342	0.049	0.147	–0.060	–0.005	0.223	–0.319	0.458
TFC	–0.054	–0.152	–0.146	–0.152	–0.445	–0.535	**0.872**	**1**	0.308	0.076	–0.153	**–0.596**	–0.342	–0.392	–0.278	–0.155	–0.323	0.013	0.479	0.112	–0.185	0.397	–0.393	0.317
CAT	–0.428	–0.565	–0.474	–0.565	–0.135	–0.137	0.189	0.308	**1**	0.354	–0.428	**–0.819**	**–0.753**	**–0.655**	–0.402	–0.435	–0.460	–0.002	–0.068	–0.317	**–0.602**	**0.733**	**–0.839**	–0.380
APX	–0.122	–0.121	0.001	–0.121	–0.361	–0.308	0.030	0.076	0.354	**1**	0.277	**–0.579**	–0.469	–0.362	–0.220	–0.192	–0.327	–0.075	–0.066	–0.158	–0.283	0.168	–0.366	0.141
GR	**0.578**	**0.595**	0.470	**0.595**	–0.018	0.037	–0.210	–0.153	–0.428	0.277	**1**	0.243	0.096	0.434	0.190	0.500	0.438	0.294	0.043	0.107	0.107	–0.519	0.377	0.465
SOD	0.430	0.576	0.272	0.576	0.396	0.413	–0.419	**–0.596**	**–0.819**	**–0.579**	0.243	**1**	**0.762**	**0.817**	**0.628**	0.554	**0.690**	0.239	–0.256	0.077	0.412	**–0.744**	**0.801**	0.173
FAA	0.331	**0.642**	0.560	**0.642**	–0.072	–0.056	–0.114	–0.342	**–0.753**	–0.469	0.096	**0.762**	**1**	**0.712**	0.428	0.300	0.335	–0.117	0.074	0.437	**0.796**	**–0.593**	**0.862**	0.171
PP	**0.740**	**0.822**	0.256	**0.822**	0.119	0.140	–0.234	–0.392	**–0.655**	–0.362	0.434	**0.817**	**0.712**	**1**	**0.834**	**0.785**	**0.866**	0.481	–0.260	–0.043	0.278	**–0.872**	**0.778**	0.478
Starch	**0.658**	0.499	–0.074	0.499	0.292	0.296	–0.264	–0.278	–0.402	–0.220	0.190	**0.628**	0.428	**0.834**	**1**	**0.825**	**0.891**	0.533	–0.222	–0.169	–0.105	**–0.702**	**0.597**	0.405
TS	**0.926**	**0.613**	–0.054	**0.613**	0.087	0.111	–0.247	–0.155	–0.435	–0.192	0.500	0.554	0.300	**0.785**	**0.825**	**1**	**0.933**	**0.747**	–0.032	–0.044	–0.149	**–0.589**	0.520	0.392
RS	**0.800**	**0.579**	–0.090	**0.579**	0.337	0.351	–0.342	–0.323	–0.460	–0.327	0.438	**0.690**	0.335	**0.866**	**0.891**	**0.933**	**1**	**0.711**	–0.263	–0.227	–0.162	**–0.732**	0.542	0.387
K+	**0.659**	0.459	–0.457	0.459	–0.067	–0.051	0.049	0.013	–0.002	–0.075	0.294	0.239	–0.117	0.481	0.533	**0.747**	**0.711**	**1**	–0.409	–0.527	–0.541	–0.270	–0.024	0.328
Na+	0.072	–0.172	0.357	–0.172	–0.184	–0.196	0.147	0.479	–0.068	–0.066	0.043	–0.256	0.074	–0.260	–0.222	–0.032	–0.263	–0.409	**1**	**0.850**	0.302	0.367	0.192	–0.167
Na+/K+	0.082	0.089	**0.702**	0.089	–0.166	–0.153	–0.060	0.112	–0.317	–0.158	0.107	0.077	0.437	–0.043	–0.169	–0.044	–0.227	–0.527	**0.850**	**1**	**0.685**	0.170	0.475	–0.177
EL	0.035	0.427	**0.832**	0.427	–0.237	–0.233	–0.005	–0.185	**–0.602**	–0.283	0.107	0.412	**0.796**	0.278	–0.105	–0.149	–0.162	–0.541	0.302	**0.685**	**1**	–0.278	**0.637**	0.100
RW	–0.550	**–0.680**	–0.279	**–0.680**	–0.226	–0.227	0.223	0.397	**0.733**	0.168	–0.519	**–0.744**	**–0.593**	**–0.872**	**–0.702**	**–0.589**	**–0.732**	–0.270	0.367	0.170	–0.278	**1**	**–0.681**	**–0.616**
MSI	0.498	**0.632**	**0.603**	**0.632**	0.159	0.189	–0.319	–0.393	**–0.839**	–0.366	0.377	**0.801**	**0.862**	**0.778**	**0.597**	0.520	0.542	–0.024	0.192	0.475	**0.637**	**–0.681**	**1**	0.267
TC	0.485	**0.626**	0.249	**0.626**	–0.248	–0.308	0.458	0.317	–0.380	0.141	0.465	0.173	0.171	0.478	0.405	0.392	0.387	0.328	–0.167	–0.177	0.100	**–0.616**	0.267	**1**

Values in bold are different from 0 with a significance level alpha=0.05

p-values (Pearson):

Variables	Proline	ROS	MDA	H_2O_2	ABTS	DPPH	TPC	TFC	CAT	APX	GR	SOD	FAA	PP	Starch	TS	RS	K^+	Na^+	Na^+/K^+	EL	RW	MSI	TC
Proline	**0**	**0.009**	0.723	**0.009**	0.539	0.587	0.682	0.867	0.165	0.705	**0.049**	0.163	0.294	**0.006**	**0.020**	**<0.0001**	**0.002**	**0.020**	0.825	0.800	0.913	0.064	0.100	0.110
ROS	**0.009**	**0**	0.100	**<0.0001**	0.304	0.351	0.790	0.636	0.056	0.708	**0.041**	0.050	**0.025**	**0.001**	0.099	**0.034**	**0.049**	0.133	0.592	0.783	0.166	**0.015**	**0.027**	**0.029**
MDA	0.723	0.100	**0**	0.100	0.672	0.724	0.834	0.651	0.119	0.998	0.123	0.393	0.058	0.421	0.819	0.868	0.782	0.136	0.255	**0.011**	**0.001**	0.380	**0.038**	0.435
H2O2	**0.009**	**<0.0001**	0.100	**0**	0.304	0.351	0.790	0.636	0.056	0.708	**0.041**	0.050	**0.025**	**0.001**	0.099	**0.034**	**0.049**	0.133	0.592	0.783	0.166	**0.015**	**0.027**	**0.029**
ABTS	0.539	0.304	0.672	0.304	**0**	**<0.0001**	0.052	0.147	0.676	0.249	0.956	0.203	0.824	0.713	0.358	0.788	0.284	0.836	0.567	0.606	0.459	0.479	0.623	0.436
DPPH	0.587	0.351	0.724	0.351	**<0.0001**	**0**	**0.019**	0.073	0.671	0.329	0.909	0.182	0.863	0.663	0.351	0.731	0.263	0.876	0.541	0.634	0.466	0.478	0.556	0.330
TPC	0.682	0.790	0.834	0.790	0.052	**0.019**	**0**	**0.000**	0.557	0.927	0.513	0.176	0.724	0.464	0.407	0.438	0.276	0.879	0.649	0.853	0.988	0.487	0.312	0.135
TFC	0.867	0.636	0.651	0.636	0.147	0.073	**0.000**	**0**	0.329	0.814	0.635	**0.041**	0.277	0.207	0.381	0.631	0.306	0.968	0.115	0.728	0.564	0.202	0.207	0.315
CAT	0.165	0.056	0.119	0.056	0.676	0.671	0.557	0.329	**0**	0.258	0.165	**0.001**	**0.005**	**0.021**	0.195	0.157	0.132	0.996	0.833	0.315	**0.038**	**0.007**	**0.001**	0.223
APX	0.705	0.708	0.998	0.708	0.249	0.329	0.927	0.814	0.258	**0**	0.383	**0.048**	0.124	0.247	0.491	0.549	0.300	0.816	0.840	0.623	0.372	0.602	0.242	0.663
GR	**0.049**	**0.041**	0.123	**0.041**	0.956	0.909	0.513	0.635	0.165	0.383	**0**	0.447	0.767	0.158	0.553	0.098	0.154	0.354	0.895	0.742	0.740	0.084	0.227	0.128

SOD	0.163	0.050	0.393	0.050	0.203	0.182	0.176	**0.041**	**0.001**	**0.048**	0.447	**0**	**0.004**	**0.001**	**0.029**	0.062	**0.013**	0.455	0.422	0.813	0.183	**0.006**	**0.002**	0.590
FAA	0.294	**0.025**	0.058	**0.025**	0.824	0.863	0.724	0.277	**0.005**	0.124	0.767	**0.004**	**0**	**0.009**	0.165	0.343	0.288	0.718	0.819	0.156	**0.002**	**0.042**	**0.000**	0.596
PP	**0.006**	**0.001**	0.421	**0.001**	0.713	0.663	0.464	0.207	**0.021**	0.247	0.158	**0.001**	**0.009**	**0**	**0.001**	**0.002**	**0.000**	0.114	0.414	0.894	0.382	**0.000**	**0.003**	0.116
Starch	**0.020**	0.099	0.819	0.099	0.358	0.351	0.407	0.381	0.195	0.491	0.553	**0.029**	0.165	**0.001**	**0**	**0.001**	**0.000**	0.074	0.488	0.600	0.746	**0.011**	**0.040**	0.191
TS	**<0.0001**	**0.034**	0.868	**0.034**	0.788	0.731	0.438	0.631	0.157	0.549	0.098	0.062	0.343	**0.002**	**0.001**	**0**	**<0.0001**	**0.005**	0.921	0.891	0.644	**0.044**	0.083	0.208
RS	**0.002**	**0.049**	0.782	**0.049**	0.284	0.263	0.276	0.306	0.132	0.300	0.154	**0.013**	0.288	**0.000**	**0.000**	**<0.0001**	**0**	**0.010**	0.408	0.479	0.615	**0.007**	0.069	0.215
K+	**0.020**	0.133	0.136	0.133	0.836	0.876	0.879	0.968	0.996	0.816	0.354	0.455	0.718	0.114	0.074	**0.005**	**0.010**	**0**	0.187	0.078	0.069	0.396	0.942	0.299
Na+	0.825	0.592	0.255	0.592	0.567	0.541	0.649	0.115	0.833	0.840	0.895	0.422	0.819	0.414	0.488	0.921	0.408	0.187	**0**	**0.000**	0.339	0.241	0.549	0.604
Na+/K+	0.800	0.783	**0.011**	0.783	0.606	0.634	0.853	0.728	0.315	0.623	0.742	0.813	0.156	0.894	0.600	0.891	0.479	0.078	**0.000**	**0**	**0.014**	0.598	0.119	0.582
EL	0.913	0.166	**0.001**	0.166	0.459	0.466	0.988	0.564	**0.038**	0.372	0.740	0.183	**0.002**	0.382	0.746	0.644	0.615	0.069	0.339	**0.014**	**0**	0.382	**0.026**	0.758
RW	0.064	**0.015**	0.380	**0.015**	0.479	0.478	0.487	0.202	**0.007**	0.602	0.084	**0.006**	**0.042**	**0.000**	**0.011**	**0.044**	**0.007**	0.396	0.241	0.598	0.382	**0**	**0.015**	**0.033**
MSI	0.100	**0.027**	**0.038**	**0.027**	0.623	0.556	0.312	0.207	**0.001**	0.242	0.227	**0.002**	**0.000**	**0.003**	**0.040**	0.083	0.069	0.942	0.549	0.119	**0.026**	**0.015**	**0**	0.402
TC	0.110	**0.029**	0.435	**0.029**	0.436	0.330	0.135	0.315	0.223	0.663	0.128	0.590	0.596	0.116	0.191	0.208	0.215	0.299	0.604	0.582	0.758	**0.033**	0.402	**0**

Coefficients of determination (Pearson):

Variables	Proline	ROS	MDA	H_2O_2	ABTS	DPPH	TPC	TFC	CAT	APX	GR	SOD	FAA	PP	Starch	TS	RS	K^+	Na^+	Na^+/K^+	EL	RW	MSI	TC
Proline	**1**	0.510	0.013	0.510	0.039	0.031	0.018	0.003	0.183	0.015	0.334	0.185	0.109	0.548	0.433	0.857	0.640	0.434	0.005	0.007	0.001	0.302	0.248	0.235
ROS	0.510	**1**	0.248	1.000	0.105	0.087	0.007	0.023	0.319	0.015	0.354	0.331	0.412	0.676	0.249	0.376	0.335	0.211	0.030	0.008	0.183	0.463	0.400	0.392
MDA	0.013	0.248	**1**	0.248	0.019	0.013	0.005	0.021	0.225	0.000	0.221	0.074	0.314	0.066	0.006	0.003	0.008	0.208	0.127	0.493	0.692	0.078	0.364	0.062
H2O2	0.510	1.000	0.248	**1**	0.105	0.087	0.007	0.023	0.319	0.015	0.354	0.331	0.412	0.676	0.249	0.376	0.335	0.211	0.030	0.008	0.183	0.463	0.400	0.392
ABTS	0.039	0.105	0.019	0.105	**1**	0.979	0.327	0.198	0.018	0.130	0.000	0.157	0.005	0.014	0.085	0.008	0.114	0.004	0.034	0.028	0.056	0.051	0.025	0.062
DPPH	0.031	0.087	0.013	0.087	0.979	**1**	0.436	0.286	0.019	0.095	0.001	0.171	0.003	0.020	0.087	0.012	0.123	0.003	0.038	0.023	0.054	0.052	0.036	0.095
TPC	0.018	0.007	0.005	0.007	0.327	0.436	**1**	0.760	0.036	0.001	0.044	0.175	0.013	0.055	0.070	0.061	0.117	0.002	0.021	0.004	0.000	0.050	0.102	0.210
TFC	0.003	0.023	0.021	0.023	0.198	0.286	0.760	**1**	0.095	0.006	0.023	0.356	0.117	0.154	0.077	0.024	0.104	0.000	0.229	0.013	0.034	0.157	0.154	0.101
CAT	0.183	0.319	0.225	0.319	0.018	0.019	0.036	0.095	**1**	0.126	0.183	0.670	0.567	0.429	0.162	0.189	0.212	0.000	0.005	0.101	0.363	0.537	0.704	0.144
APX	0.015	0.015	0.000	0.015	0.130	0.095	0.001	0.006	0.126	**1**	0.077	0.336	0.220	0.131	0.049	0.037	0.107	0.006	0.004	0.025	0.080	0.028	0.134	0.020
GR	0.334	0.354	0.221	0.354	0.000	0.001	0.044	0.023	0.183	0.077	**1**	0.059	0.009	0.189	0.036	0.250	0.192	0.086	0.002	0.011	0.011	0.269	0.142	0.216
SOD	0.185	0.331	0.074	0.331	0.157	0.171	0.175	0.356	0.670	0.336	0.059	**1**	0.581	0.667	0.395	0.307	0.476	0.057	0.066	0.006	0.170	0.553	0.642	0.030
FAA	0.109	0.412	0.314	0.412	0.005	0.003	0.013	0.117	0.567	0.220	0.009	0.581	**1**	0.506	0.184	0.090	0.112	0.014	0.005	0.191	0.634	0.352	0.743	0.029
PP	0.548	0.676	0.066	0.676	0.014	0.020	0.055	0.154	0.429	0.131	0.189	0.667	0.506	**1**	0.695	0.617	0.750	0.231	0.068	0.002	0.077	0.760	0.606	0.228
Starch	0.433	0.249	0.006	0.249	0.085	0.087	0.070	0.077	0.162	0.049	0.036	0.395	0.184	0.695	**1**	0.681	0.793	0.284	0.049	0.028	0.011	0.493	0.356	0.164
TS	0.857	0.376	0.003	0.376	0.008	0.012	0.061	0.024	0.189	0.037	0.250	0.307	0.090	0.617	0.681	**1**	0.871	0.559	0.001	0.002	0.022	0.347	0.271	0.154
RS	0.640	0.335	0.008	0.335	0.114	0.123	0.117	0.104	0.212	0.107	0.192	0.476	0.112	0.750	0.793	0.871	**1**	0.506	0.069	0.051	0.026	0.536	0.293	0.149
K+	0.434	0.211	0.208	0.211	0.004	0.003	0.002	0.000	0.000	0.006	0.086	0.057	0.014	0.231	0.284	0.559	0.506	**1**	0.167	0.278	0.293	0.073	0.001	0.107
Na+	0.005	0.030	0.127	0.030	0.034	0.038	0.021	0.229	0.005	0.004	0.002	0.066	0.005	0.068	0.049	0.001	0.069	0.167	**1**	0.723	0.091	0.134	0.037	0.028
Na+/K+	0.007	0.008	0.493	0.008	0.028	0.023	0.004	0.013	0.101	0.025	0.011	0.006	0.191	0.002	0.028	0.002	0.051	0.278	0.723	**1**	0.469	0.029	0.225	0.031
EL	0.001	0.183	0.692	0.183	0.056	0.054	0.000	0.034	0.363	0.080	0.011	0.170	0.634	0.077	0.011	0.022	0.026	0.293	0.091	0.469	**1**	0.077	0.406	0.010
RW	0.302	0.463	0.078	0.463	0.051	0.052	0.050	0.157	0.537	0.028	0.269	0.553	0.352	0.760	0.493	0.347	0.536	0.073	0.134	0.029	0.077	**1**	0.464	0.380
MSI	0.248	0.400	0.364	0.400	0.025	0.036	0.102	0.154	0.704	0.134	0.142	0.642	0.743	0.606	0.356	0.271	0.293	0.001	0.037	0.225	0.406	0.464	**1**	0.071
TC	0.235	0.392	0.062	0.392	0.062	0.095	0.210	0.101	0.144	0.020	0.216	0.030	0.029	0.228	0.164	0.154	0.149	0.107	0.028	0.031	0.010	0.380	0.071	**1**

Table S2. Different abiotic stress conditions in which peanut plants were subjected for 48 h.

S. No.	Stress conditions	Code[#]	Media and supplement
1.	Control (no stress)	Control	MSB (pH 5.8)
2..	Salinity	Salinity	MSB + 200 Mm NaCl
3.	Drought	Drought	MSB + 10% PEG
4.	Heat	Heat	MSB + 45°C
5.	Cold	Cold	MSB + 4°C
6.	Salinity and Drought	S–D	MSB + 200 mM NaCl +10% PEG
7.	Salinity and Heat	S–H	MSB + 200 mM NaCl + 45°C
8.	Salinity and Cold	S–C	MSB + 200 mM NaCl + 4°C
9.	Drought and Heat	D–H	MSB +10% PEG + 45°C
10.	Drought and Cold	D–C	MSB +10% PEG + 4°C
11.	Salinity, Drought, and Heat	S–D–H	MSB + 200 mM NaCl +10% PEG + 45°C
12.	Salinity, Drought, and Cold	S–D–C	MSB + 200 mM NaCl +10% PEG + 4°C

Codes used in the study
MSB: Murashige and Skoog Basal Medium
PEG: Polyethylene glycol

Table S3. Primers and PCR conditions used for the expression analysis of antioxidant encoding genes using real time PCR.

Genes	Primer code	Orientation	Sequence (5′-3′)	PCR conditions^
Ascorbate peroxidase	*Ah*APX–F	Forward	TGCTGGAACTTTTGATGTGG	1 cycle<br>95°C, 5 min
	*Ah*APX–R	Reverse	AACTACACCGGCCAACTG	
Catalase	*Ah*CAT–F	Forward	TTTTACACCAGAGAGGGTAACT	40 cycles<br>95°C, 10 s<br>60°C, 30 s
	*Ah*CAT–R	Reverse	AGGATCCTCCAATTCTCCTGG	
Superoxide dismutase	*Ah*SOD–F	Forward	CAGTTCTTAGCAGCAGTGAG	
	*Ah*SOD–R	Reverse	GGAACCCATGAAGACCAG	
Actin	*Ah*ACT–F	Forward	CGGGATGGAATCTCCTGGA	Melt curve analysis
	*Ah*ACT–R	Reverse	CATGCTACTCGGTGCCAATG	

^ PCR conditions are same for all
